# Parafoveal preview differentially modulates word frequency and contextual predictability effects during reading

**DOI:** 10.1167/jov.26.2.13

**Published:** 2026-02-19

**Authors:** Sara C. Sereno, Christopher J. Hand, Aisha Shahid, Bo Yao

**Affiliations:** 1 School of Psychology & Neuroscience, University of Glasgow, Glasgow, UK; 2 School of Education, University of Glasgow, Glasgow, UK; 3 The Open University, Edinburgh, UK; 4 Department of Psychology, Lancaster University, Lancaster, UK

**Keywords:** word frequency, contextual predictability, parafoveal preview, eye movements, reading

## Abstract

Despite more than five decades of research into eye movements in reading, questions remain about the relationship between lower-level lexical and higher-level semantic factors. We explored the simultaneous effects of word frequency (lower, higher), contextual predictability (lower, higher), and parafoveal preview (valid, invalid) on the processing of target words embedded in short passages of text. Using a repeated-measures design, 80 participants read 240 two-line passages, each containing a four- or five-letter target word. Corpus-based word frequencies and Cloze predictabilities were used as continuous variables in Bayesian mixed-effect analyses of fixation time and skipping measures. Key findings included robust main effects of frequency, predictability, and preview validity, as well as two-way interactions between Frequency × Preview in gaze duration, and Predictability × Preview in gaze duration and skipping. Frequency effects on gaze duration were greater under invalid preview conditions, suggesting that higher-frequency words facilitate corrective processing when preview is misleading. Predictability effects on gaze duration and skipping were enhanced under valid preview, indicating that contextual facilitation depends on coherent parafoveal input. No interaction was observed between frequency and predictability nor a three-way interaction, supporting the view that lexical access and contextual integration operate via distinct mechanisms. These findings highlight the critical role of parafoveal information in shaping the expression of lexical and contextual influences during reading.

## Introduction

Over the past 50 years, the principal advancements in understanding reading—the visual comprehension of language—have come from investigations that analyze eye movement behavior, namely, the position, duration, and sequence of eye fixations in text (for reviews, see Rayner, 1998; Rayner, 2009). Kowler (2011) contextualized reading within the larger science of eye movements, underscoring the importance of understanding eye movements as both a window into and a mechanism of cognitive processing. Aside from the physiological constraints of acuity, oculomotor programming, and processing speed, the properties of language itself, from its orthography to higher-level semantics, typically leave discernible “eye-prints” in the data. The current study examined the role of parafoveal preview in the recognition of high- and low-frequency words occurring in semantically biasing and neutral contexts.

### Word frequency and contextual predictability

It is well established that, after accounting for the effects of word length (e.g., Rayner, Sereno, & Raney, 1996), the two key variables that influence fixation time on a word in reading are its frequency of occurrence and its predictability from the prior context (e.g., Rayner 1998; Rayner, 2009; for a recent review, see Sereno, Hand, Shahid, Yao, & O'Donnell, 2018). Research investigations into word frequency have consistently demonstrated that high-frequency (HF) words are fixated for shorter durations and skipped more often than low-frequency (LF) words (e.g., Hand, Miellet, O'Donnell, & Sereno, 2010; Hand, O'Donnell, & Sereno, 2012; Inhoff & Rayner, 1986; Just & Carpenter, 1980; Kennedy, Pynte, Murray, & Paul, 2013; Kliegl, Grabner, Rolfs, & Engbert, 2004; Miellet, Sparrow, & Sereno, 2007; Rayner & Duffy, 1986; Rayner et al., 1996; Rayner, Ashby, Pollatsek, & Reichle, 2004; Scott, O'Donnell, & Sereno, 2012; Sereno, 1992; Sereno, O'Donnell, & Rayner, 2006; Sereno, Pacht, & Rayner, 1992; Sereno et al., 2018; Sereno & Rayner, 2000; Slattery, Pollatsek, & Rayner, 2007). Likewise, numerous studies examining contextual predictability have shown that high predictability (HP) words are fixated for shorter durations and skipped more often than low predictability (LP) words (e.g., Balota, Pollatsek, & Rayner, 1985; Ehrlich & Rayner, 1981; Hand et al., 2010; Hand et al., 2012; Kennedy et al., 2013; Kliegl et al., 2004; McDonald & Shillcock, 2003; Miellet et al., 2007; Morris, 1994; Rayner et al., 2004; Rayner & Well, 1996; Sereno et al., 2018; Veldre & Andrews, 2018; for a review, see Staub, 2015).

Only a handful of eye movement studies have investigated the simultaneous effects of both frequency and predictability in reading (e.g., Hand et al., 2010; Hand et al., 2012; Kennedy et al., 2013; Kliegl et al., 2004; Miellet et al., 2007; Rayner et al., 2004; Sereno et al., 2018; Staub & Goddard, 2019). In general, these studies have demonstrated additive effects of frequency and predictability. However, interactive findings have been reported most often when alternative measures have been used, such as word naming or lexical decision paradigms (e.g., Stanovich & West, 1983; West & Stanovich, 1982) and event-related potentials (e.g., Dambacher, Kliegl, Hofmann, & Jacobs, 2006; Dambacher et al., 2012; Kretzschmar, Schlesewsky, & Staub, 2015; Sereno, Brewer, & O'Donnell, 2003; Sereno, Hand, Shahid, Mackenzie, & Leuthold, 2019; van Petten & Kutas, 1990; but cf., Penolazzi, Hauk, & Pulvermüller, 2007, for additive effects). Notably, in such studies, the target word typically appears on screen as an isolated event and is not seen parafoveally as would be the case in normal reading. Nevertheless, some eye movement studies have demonstrated a frequency-predictability interaction (e.g., launch site analysis in Hand et al., 2010; Inhoff, 1984; Sereno et al., 2018). Overall, there remains a degree of uncertainty regarding the exact nature of frequency-predictability effects—the conditions under which alternative patterns of effects emerge and what this implies in terms of temporal and representational constraints on lexical access.

A central theoretical issue concerns how frequency and predictability exert their influence during word recognition and whether these effects are independent or interactive. Modular accounts (e.g., Fodor, 1983) posit that lexical access proceeds in a strictly bottom-up fashion, with contextual information affecting processing only after word identification. By contrast, interactive frameworks (e.g., such as McClelland, 1987) assume that contextual constraints can directly shape lexical access by pre-activating word candidates, leading to frequency-predictability interactions at early stages of processing. More recent probabilistic approaches, such as Levy's (2008) surprisal theory, formalize predictability in terms of information-theoretic expectations, with processing cost inversely related to a word's conditional probability in context. Converging evidence from electrophysiology supports this interactive view, showing that frequency effects are associated with early stages of lexical access, while predictability effects can arise even earlier and persist through later semantic integration (e.g., Kretzschmar et al., 2015; Sereno et al., 2003; Sereno et al., 2019). Thus the relative timings and loci of frequency and predictability effects provide critical leverage for distinguishing between theoretical accounts. Importantly, parafoveal preview is central to this debate: access to upcoming visual information allows contextual constraints to be deployed rapidly, whereas in the absence of valid preview, readers rely more heavily on frequency-based lexical activation.

### The role of parafoveal preview

Early eye movement studies recognized that the amount and type of parafoveal preview of an upcoming target word plays a key role in its subsequent identification (e.g., McConkie & Rayner, 1975; Rayner, 1975). Eye movement research has used display change techniques such as the moving window paradigm (McConkie & Rayner, 1975), the boundary technique (Rayner, 1975), and, more recently, parafoveal magnification (Miellet, O'Donnell, & Sereno, 2009; Yao, Hand, Miellet, & Sereno, 2025) to determine the type and limits of information obtained parafoveally. In general, such research has demonstrated that a target's (eventual) fixation time depends not only on the eyes’ eccentricity to the target, but also on the target's orthographic, phonological, and semantic properties (for reviews, see Balota & Rayner, 1991; Schotter, Angele, & Rayner, 2012).

The role of parafoveal processing in understanding the nature of frequency-predictability effects is essential to revealing the underlying processing constraints for models of reading. However, research that has simultaneously manipulated frequency, predictability, and preview has been relatively limited. Moreover, it is difficult to generalize across such studies, given the variability with which the parafoveal preview factor has been instantiated. A variety of parafoveal previews have been used to determine the level of preview benefit conferred to the eventual processing of the target (for a review, see Schotter et al., 2012). These include preview-target relationships, respectively, spanning lower- to higher-level lexical attributes such as the following: case change (e.g., *wOrD-WoRd*; McConkie & Zola, 1979), transposed letters (e.g., *jugde-judge*; Johnson, Perea, & Rayner, 2007), orthographic neighbors (e.g., *sweet-sleet*; Williams, Perea, Pollatsek, & Rayner, 2006), homophones (e.g., *bare-bear*; Pollatsek, Lesch, Morris, & Rayner, 1992), and semantic associates (e.g., *cake-pies*; Balota et al., 1985).

Balota et al. (1985) was one of the first studies using the boundary technique to systematically examine parafoveal processing and contextual predictability. Although they did not manipulate frequency, their use of multiple preview conditions was influential in later research. A single sentence frame (e.g., *Since the wedding was today, the baker rushed the ____ to the reception.*) accommodated either an HP (e.g., *cake*) or LP (e.g., *pies*) word. Parafoveal previews for the HP and LP targets, respectively, were denoted as follows: identical (*cake*, *pies*); visually similar (*cahc*, *picz*), semantically related (*pies*, *cake*), visually different (*picz*, *cahc*), or anomalous (*bomb*, *bomb*). Balota et al. (1985) found that greater parafoveal benefit was obtained when previews were identical or visually similar to upcoming HP targets.

There are a few aspects of the Balota et al. (1985) study that merit further discussion as they raise issues relevant to the current study. First, the condition name of “visually similar” in Balota et al. (1985) is a bit of a misnomer as the first two letters of the four-letter word were identical, not similar. Earlier, Rayner, Well, Pollatsek, and Bertera (1982) had demonstrated that when the first three letters of a parafoveal preview were identical to those of the (eventual) target—with the remaining letters of the preview replaced by letters visually similar to the target—reading rate was only slightly impaired compared to when the preview was identical to the target (i.e., the valid preview condition). Thus it would be more accurate to interpret Balota et al.’s visually similar preview effects as ones based on having identical word-initial letters.

A second notable aspect of Balota et al.’s (1985) study is the use of illegal letter combinations (in English) in the visually similar and dissimilar preview conditions (e.g., -*hc*, -*cz*), which could influence attentional allocation. In their fast priming paradigm, Sereno and Rayner (1992) had originally used random letter string previews (e.g., *gzsd*, generated from the sequentially prior letters in the alphabet with reference to the eventual target, *hate*). To decrease the disruptive effects of such previews, Sereno (1995) instead used random letter strings generated from position-based letter probabilities (Mayzner & Tresselt, 1965) that were more word-like (e.g., *mirt*). Others have also suggested that previews that are relatively orthographically regular and pronounceable are less likely to attract parafoveal awareness, resulting in less subsequent foveal costs (Angele, Slattery, & Rayner, 2016; Reingold, Reichle, Glaholt, & Sheridan, 2012; Sereno & Rayner, 2000).

A final aspect of the Balota et al. (1985) study concerns the quality of the text displayed. Hand et al. (2012) discussed a potential artefact that may have been introduced by the relatively low-resolution dot-matrix font presented on a CRT that was used in similar past studies (e.g., Lima & Inhoff, 1985). They suggested that certain patterns of results may be explained in part by neuroimaging research, for example, from Kveraga, Boshyan, and Bar (2007), who demonstrated that lower spatial resolution (blurred) input can lead to faster top-down processing via magnocellular pathways. Gagl, Hawelka, Richlan, Schuster, and Hutzler (2014) implemented a range of parafoveal previews that were identical to the target, but visually degraded across some or all letter positions. Although they did not examine interactions with contextual predictability, they did find greater parafoveal preview benefit from constraining initial letter sequences, in contrast to Lima and Inhoff's (1985) findings but supportive of Hand et al.’s (2012).

Two recent studies that have investigated the frequency-predictability interaction with valid and invalid parafoveal previews are Sereno et al. (2018) and Staub and Goddard (2019). Sereno et al.’s (2018) targets were four- to eight-letter LF and HF length-matched words (*M_length_* = 5.88 characters) presented in the second sentence of two-line passages. Three predictability levels—low, medium, and high predictability (LP, MP, HP)—were determined by pre-tested Cloze values on individual targets, with means of 0.01, 0.52, and 0.96 for LF words and 0.01, 0.56, and 0.97 for HF words, respectively. For purposes of comparison, it is important to note that MP in this study is most consistent with what is labeled “HP” in other studies (typically, where there are only LP and HP levels of predictability; e.g., Hand et al., 2010; Rayner et al., 2004); the HP condition of Sereno et al. (2018) represents Cloze values that are relatively higher and more constrained than most studies. There were 25 words in each of the six frequency-predictability conditions. Within and across both the LF and HF conditions, different length- and frequency-matched target words were utilized in each of the LP, MP, and HP conditions. Thus there was a total of 150 distinct target words and all participants read all items. Sereno et al.’s (2018) preview manipulation was instantiated across two experiments using different participants. With valid previews (Experiment 1), they found main effects of frequency and predictability, as well as a frequency-predictability interaction in early fixation time measures. Invalid previews comprised orthographically regular pronounceable pseudowords having the same body shape as their eventual targets in terms of ascending, descending, and inline characters, and without any position-based overlapping letters (e.g., *gron* as the preview for the target *peas*). When previews were invalid (Experiment 2), although there were main effects of frequency and predictability, there was no evidence of an interaction. The between-experiment analysis revealed a preview-predictability interaction in early fixation time measures: when preview was valid, both MP and HP targets were facilitated relative to LP targets; when preview was invalid, only HP targets were facilitated (MP and LP targets did not differ).

Staub and Goddard's (2019) Experiment 1 manipulated frequency, predictability, and preview. Their Experiment 1 used 80 of the 160 items from Kretzschmar et al. (2015). Staub and Goddard's targets were 20 LF (*M_length_* = 5.25 characters) and 20 HF (*M_length_* = 4.25 characters) words presented in single-line sentences. LP and HP levels were determined by pretested Cloze values on individual targets, with means of 0.004 and 0.69 for LF words and 0.007 and 0.84 for HF words, respectively. Their invalid previews were semantically anomalous words, but having the same word shape as their eventual targets (e.g., *backs-tonic*, *bear-tree*). Each target word was read twice (as LP-valid and as HP-invalid, or as LP-invalid and as HP-valid). There were 10 items in each of the eight frequency-predictability-preview conditions. With valid previews, similar to Sereno et al. (2018), there were main effects of frequency and predictability. With invalid (semantically anomalous) previews, in contrast to Sereno et al. (2018), only the frequency effect was significant. In partial contrast to Sereno et al. (2018), Staub and Goddard found no evidence of a frequency-predictability interaction with either valid or invalid previews. Although Staub and Goddard's Experiment 2 did not manipulate frequency, they did manipulate predictability (LP, HP) and, in addition to valid and invalid (semantically anomalous) previews, they included a second invalid preview condition of random letters that were non-pronounceable, orthographically irregular consonant strings. Both invalid previews maintained the same word shape as the eventual target (e.g., *color* or *wmlmn* for the target *voice*). As in their Experiment 1, the effect of predictability disappeared when previews were invalid.

Using an alternative approach, Hand et al. (2010) investigated the role of parafoveal preview in the frequency-predictability interaction, but without manipulating the validity (i.e., valid/invalid) of the preview. Instead, they used launch distance (i.e., the distance from the pre-target fixation to the target word) as a *post hoc* proxy measure to index the extent of parafoveal processing, with near, middle, and far launch sites (1–3, 4–6, and 7–9 characters, respectively), all within the perceptual span (i.e., the region of text from which useful information can be extracted; McConkie & Rayner, 1975). Because visual acuity drops off as a function of retinal eccentricity (e.g., Anstis, 1974), Hand et al.’s (2010) approach assumes that launch distance is negatively associated with the degree of parafoveal preprocessing. While invalid previews are meant to prevent parafoveal preprocessing of the target, Hand et al. (2010) argued that any type of invalid preview introduces an incorrect stimulus that is, by its very nature, disruptive. Although manipulating preview via launch distance is not without its limitations (see, e.g., Slattery, Staub, & Rayner, 2012), this alternative approach to investigating preview has the advantages of not introducing a display change and an incorrect stimulus. Hand et al. (2010) reported significant interactive frequency-predictability findings in both near and middle launch distances. As these effects were antagonistic (with greater predictability effects for LF words at near distances, but for HF words at middle distances), the overall result was an additive pattern for the frequency-predictability interaction.

Taken together, these studies converge in showing that parafoveal preview is central to understanding how frequency and predictability shape word processing during reading, but they differ in the precise conditions under which effects emerge. Balota et al. (1985), while not manipulating target frequency, demonstrated that contextual predictability effects were contingent on accurate or partially accurate previews, underscoring the critical role of parafoveal information in enabling contextual facilitation. Sereno et al. (2018) reported a frequency-predictability interaction with valid but not invalid previews, suggesting an early locus of contextual constraint when parafoveal information is available. Staub and Goddard (2019) found persistent effects of frequency, but predictability effects only with valid previews, consistent with the view that frequency and predictability operate as separable sources of influence. Finally, Hand et al. (2010) used launch distance as an index of parafoveal processing (rather than preview validity) and showed that the balance between additive and interactive effects of frequency and predictability depended on the quality of the preview. Collectively, these studies highlight that both lexical and contextual effects are tightly bound to the availability and quality of parafoveal preview.

### The current study

The current study examined the frequency-predictability interaction under valid (i.e., identical) and invalid preview conditions. Like Sereno et al. (2018), invalid previews were orthographically legal, pronounceable pseudowords that shared the same body shape as their eventual targets in terms of ascending, descending, and inline characters, and without any position-based overlapping letters (e.g., preview-target pairs such as *larn-dune* or *pream-grave*). Such invalid previews avoid any potential complications associated with the previews that have been used in prior studies: identical letter overlap (e.g., Balota et al., 1985); odd, non-pronounceable letter combinations (e.g., Sereno & Rayner, 1992; Staub & Goddard, 2019); or semantically anomalous words (e.g., Staub & Goddard, 2019). Our target-shaped pseudowords impeded valid preview but, at the same time, were designed to evoke the least disruption. In contrast to Sereno et al. (2018), the current study adopted alternative approaches in design and analyses. First, the same LF or HF target was presented in neutral and biasing sentence frames with either valid or invalid previews in a within-participants design (N.B. participants only read one of the four predictability/preview conditions for each LF or HF target). Second, instead of analyses of variance by factors, Bayesian ex-Gaussian mixed-effect models were used (e.g., Yao et al., 2025). These models support continuous evidence assessment via Bayes factors without relying on predefined effect size for power calculations, capture both the central tendency and skew of fixation durations, and incorporate item-specific log frequency and Cloze values to more precisely estimate the influences of word frequency and contextual predictability on fixation durations.

Based on the pattern of findings from Sereno et al. (2018) and Staub and Goddard (2019), we anticipated that there would be significant main effects of Frequency, Predictability, and Preview, with shorter fixation times when targets were higher in frequency, higher in predictability, and had valid parafoveal previews. In line with the prior studies, the only interaction we expected to find was a Predictability × Preview interaction, in which Predictability effects are attenuated under conditions of invalid preview. We did not expect to find a Frequency × Predictability interaction. Staub and Goddard (2019) did not find one and, although Sereno et al. (2018) found such an interaction, it was driven by their “ultra” HP condition (avg. Cloze of ∼0.90). The HP condition of the current experiment is more comparable to their MP condition in terms of Cloze probability. We also did not expect to find a Frequency × Preview interaction or a Frequency × Predictability × Preview interaction as neither Sereno et al. (2018) nor Staub and Goddard (2019) demonstrated such interactions. Nevertheless, the current study represents an enhanced, more rigorous adaptation of the prior studies in terms of its experimental design, stimulus control, and analytic approach.

## Method

### Sample size justification

Our study adopted a Bayesian approach for data analysis and therefore did not use power calculations to determine sample size. Bayesian inference is primarily concerned with the probability distribution of parameters given the data rather than the probability of observing the data under repeated sampling assuming the null hypothesis (Kruschke & Liddell, 2018). Our sample size was determined based on the sample sizes used in Sereno et al. (2018) and Staub and Goddard (2019) as well as the recommended number of observations for well-powered reaction time studies under the frequentist framework (Brysbaert & Stevens, 2018).

Sereno et al. (2018) implemented a Frequency (HF, LF) × Predictability (HP, MP, LP) × Preview (Valid, Invalid) design with 80 participants. They used 25 items in each of the six Frequency-Predictability conditions. Preview was manipulated between participants (Valid in Experiment 1, Invalid in Experiment 2) with 40 participants in each group. Their HF and LF words were matched on an item-by-item basis exactly for word length across Predictability conditions. Their HP, MP, and LP words were almost identical in their Cloze values across Frequency conditions. Because individual contexts were devised for each target word, any given target only appeared in one Predictability condition and was read by all participants. Staub and Goddard's (2019) Experiment 1 used a Frequency (HF, LF) × Predictability (HP, LP) × Preview (Valid, Invalid) design with 72 participants. There were 20 items per each of the eight conditions. Their HF and LF words differed in length (4.25 vs. 5.25 characters, respectively). Their HP Cloze values also differed across HF and LF conditions (0.84 vs. 0.69, respectively). In Staub and Goddard's (2019) design, in contrast to that of Sereno et al. (2018), not only did the same target appear in HP and LP contexts but also both versions were read by all participants.

It is somewhat difficult to determine the number of unique observations—in terms of number of items per condition and number of participants—in the Sereno et al. (2018) and Staub and Goddard (2019) studies. In Sereno et al. (2018), Preview was manipulated between participants. Moreover, different LF or HF words, although controlled for length and frequency, were used in the different Predictability conditions. In Staub and Goddard (2019), there were some discrepancies in word length and Cloze values across Frequency conditions and participants read target words under both Predictability conditions.

In the current experiment, we used a Frequency (HF, LF) × Predictability (HP, LP) × Preview (Valid, Invalid) within-participants design with a total of 80 participants. There were 30 items in each of the eight Frequency-Predictability-Preview conditions. HF and LF targets were matched on an item-by-item basis and HP and LP Cloze values were kept minimally different between Frequency conditions. The same HF or LF word appeared in the different Predictability and Preview conditions and participants only read each target word once. Thus we were able to achieve 2400 observations per condition (30 trials per condition × 80 participants), which is well above the minimum of 1600 necessary to detect effect sizes of 10–20 ms as recommended by Brysbaert and Stevens (2018).

### Participants

A total of 80 members of the University of Glasgow community (42 female, 38 male; *M*_age_ = 24 years) took part in the experiment. All participants were native English speaking, had normal or corrected-to-normal vision, and had not been diagnosed with any reading or learning disorder. Participants were compensated at a rate of £6 per hour. All participants provided written informed consent, and the experimental procedure was approved by the Research Ethics Committee at the University of Glasgow.

### Design and materials

We used a 2 (Frequency: HF, LF) × 2 (Predictability: HP, LP) × 2 (Preview: Valid, Invalid) repeated-measures design. Although our design was categorical, Frequency and Predictability are continuous variables. Thus, for analyses, the item-specific values of Frequency and Predictability were used in our models.

Experimental materials comprised a set of 240 two-line passages. The first “context sentence” was devised to be either biasing or neutral with respect to the target word that appeared in the second “target sentence.” Target words were either four or five characters long and were matched exactly for length across conditions. Half of the targets were HF words and half were LF words. Target word frequencies, measured in occurrences per million, were acquired from the British National Corpus, a database of 90 million written word tokens (Davies, 2004).

Target word predictability, when preceded by context sentences designed to be either HP (biasing) or LP (neutral), was determined via a Cloze task. Forty participants (27 female, 13 male; *M*_age_ = 21 years) from the University of Glasgow community took part in the Cloze task. None took part in the main reading experiment. All were native English speakers, had not been diagnosed with any reading or learning disorder, and were compensated at a rate of £6 per hour for their participation. Passages were presented up to but not including the HF or LF target word or post-target text. Participants were instructed to generate what they thought the next word of the passage should be. Responses were scored as “1” for correct responses (i.e., the targets used by the authors), “0.5” if it was the correct word but had a different grammatical inflection (e.g., “hand” instead of “hands”), and “0” for any other word. Two versions of the passages were devised: one group of 20 participants received all HF and LF target sentence onsets, with half of each frequency condition preceded by HP contexts and half preceded by LP contexts; the other group of 20 participants received the complement set of passages with the alternative mapping of predictability conditions. Average Cloze probabilities for target words for both HP and LP conditions (i.e., when preceded by biasing or neutral contexts, respectively) were calculated.

Target word specifications in terms of length, frequency, and predictability are presented in Table 1. Supplementary Materials A contains a full list of target words and their associated specifications across these dimensions.

**Table 1.** Mean specifications of target words (with *SD*s).

	HF	LF
Length	4.77 (0.42)	4.77 (0.42)
Frequency	103.79 (91.39)	7.91 (5.50)
HP Cloze	0.67 (0.24)	0.61 (0.26)
LP Cloze	0.07 (0.12)	0.05 (0.09)

*Note*: Units of measures are as follows: Length in number of characters; Frequency in occurrences per million; Cloze as a probability that the target word was correctly guessed within the given context.

Finally, the parafoveal preview of the target word—that is, what was displayed in the target word region when the eyes were to the left of this region—was either valid or invalid. Valid previews were the targets themselves. Invalid previews were orthographically legal, pronounceable pseudowords whose overall shape mimicked their targets in terms of ascending, descending, and inline characters (e.g., *shest* for *clock*). Such invalid previews were used to prevent parafoveal processing of the target on pre-target fixations while simultaneously causing as little disruption as possible compared to other invalid previews that have been used in the past. The boundary technique (Rayner, 1975; Sereno et al., 2018) was used whereby the invalid preview was replaced with the target when participants made a saccade over the last letter of the pre-target word.

Supplementary Materials B includes the complete set of 240 experimental passages (Table B1), the counterbalancing of conditions by items across the four presentation lists (Table B2) and the mean target word length, frequency, and predictability values by item groupings (Table B3).

### Apparatus

Participants’ eye movements were recorded using an EyeLink 2K eye tracker (SR Research Ltd., Mississauga, Ontario, Canada). Eye movements were sampled at 1000 Hz using corneal reflection and pupil tracking. The spatial resolution was 0.01°. EyeTrack software, developed by the University of Massachusetts, Amherst, controlled stimulus presentation (https://blogs.umass.edu/eyelab/software/). All 240 passages of text, as well as the practice trials, were presented in 14-point Bitstream Vera Sans Mono nonproportional font (black characters on a white background) using quadruple line spacing, displayed on a Dell P1130 19” flat screen monitor (1024 × 768 resolution; 150 Hz). At a viewing distance of 72 cm, approximately four characters subtended 1° of visual angle. Viewing was binocular and eye movements were recorded from the right eye. A forehead/chin rest was used to minimize head movements.

### Procedure

On arrival, participants were given an information sheet and a consent form. Participants were asked to read the passages for comprehension as if they were reading a magazine article. They were informed there were Yes/No questions after some of the passages to ensure they were paying attention. After the initial calibration and validation procedure (nine-point, full horizontal and vertical range), participants read eight practice passages that were representative of the eight experimental conditions (with five questions). This was followed by the 240 experimental trials (with 80 associated questions), presented in a random order. Participants self-paced their breaks, and calibration and validation procedures were repeated after each break and as necessary throughout the session.

Each passage began when the participant generated a stable fixation on an upper left fixation point, corresponding to the first character of text. After reading the passage, participants moved their eyes to the bottom right of the screen and pressed a button on a hand-held game controller to clear the display. On two-thirds of the trials, the next trial began. On the remaining one-third of the trials, a question first appeared with the words “No” and “Yes” below, and participants pressed the left or right button, respectively, to indicate their response. On average, participants answered 96% of the questions correctly.

The boundary paradigm was implemented on half of the trials that contained an invalid preview in the second, target sentence of the passages. On these trials, the invalid preview was displayed in the target location until the participants’ eyes traversed an invisible boundary (located after the last character of the pre-target word), when it was replaced by the target which remained on screen until the end of the trial. Display changes from preview to target were made within 6.67 ms (one refresh cycle of the 150 Hz monitor).

At the end of the experiment, participants were asked for feedback. Although many noticed changes or flickers that sometimes occurred in the text while reading, none were able to identify what they had seen. The experiment lasted approximately 50 minutes.

## Results

### Data availability

Relevant data files and R scripts can be found via the Open Science Framework (https://osf.io/6t5ps/).

### Data preprocessing

To prepare the eye-tracking data for statistical analyses, a suite of data management programs (https://blogs.umass.edu/eyelab/software/) were used. The data were first subject to common cleaning processes used in eye movement reading studies (e.g., Hand et al., 2010; Ingram & Hand, 2020; Yao, Scott, Bruce, Monteith-Hodge, & Sereno, 2024). The target region included the space before the target word and the target word itself. Individual fixations shorter than 100 ms or longer than 750 ms were eliminated (N.B. fixations less than 100 ms but within one character of another fixation were merged). Data were additionally eliminated from analyses if a blink or track loss occurred on the target word, or if the fixation on the target was either the first or the last fixation on the line. There was additional data loss on invalid preview trials when the display change occurred inappropriately—for example, the boundary was triggered prematurely because of dynamic overshoot of a pre-target saccade (e.g., Becker, 1989) or it was triggered during the target word fixation, itself, because of fixation drift or calibration error. Across all possible exclusion criteria, approximately 12% of trials were excluded from further analysis. The percentages of the remaining data for first-pass single fixation, immediate refixation, and first-pass skipping of the target were 65%, 11%, and 24%, respectively.

**Figure 1.**
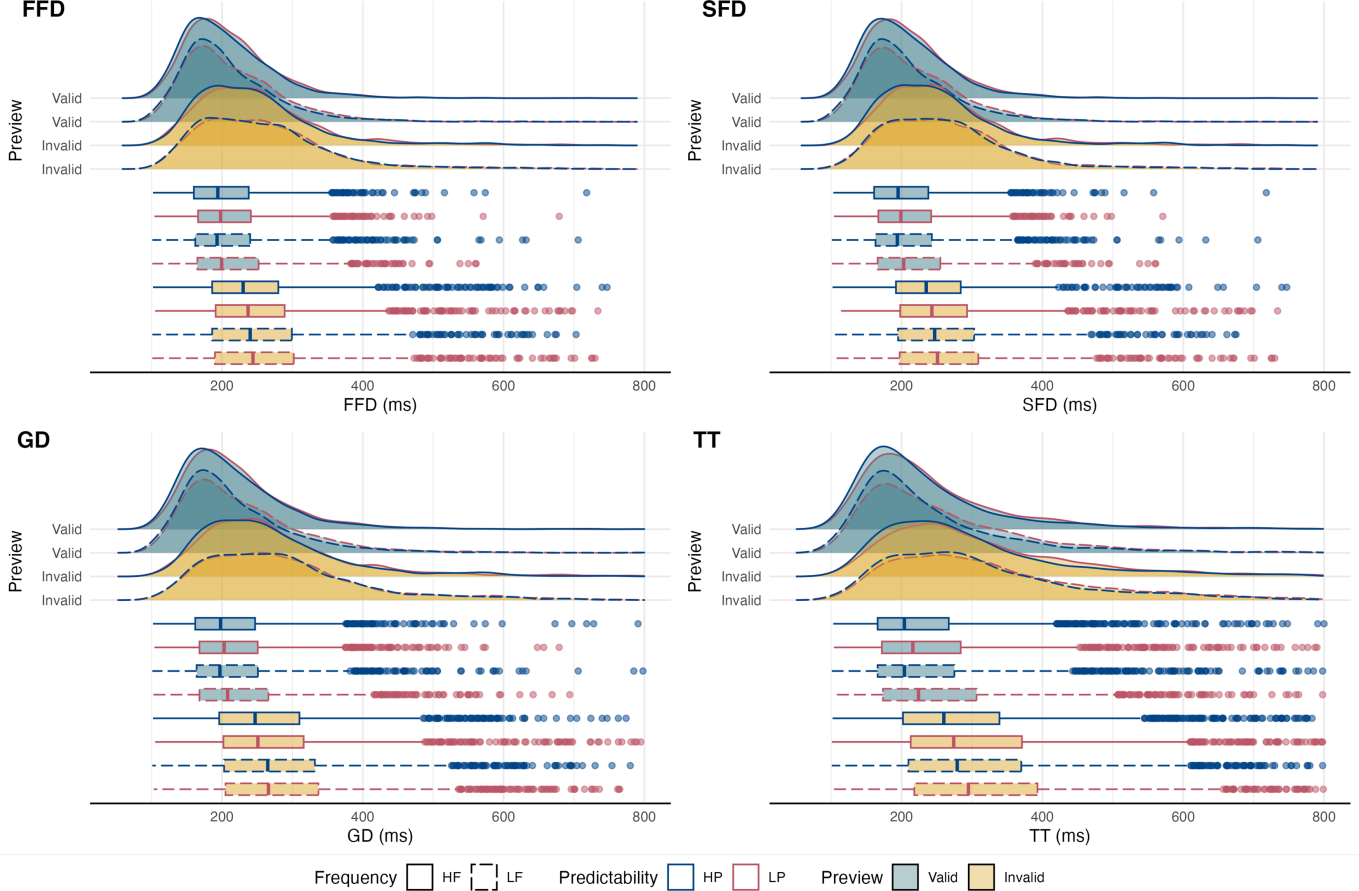
Raw distributions of fixation durations by experimental design. Fixation duration measures represented in panels as follows: FFD = top-left; SFD = top-right; GD = bottom-left; and TT = bottom-right. Frequency is categorized by line type, with HF = solid lines and LF = dashed lines. Predictability is categorized by line color, with HP = blue and LP = red. Preview is categorized by fill color, with Valid = teal and Invalid = yellow. Valid = valid preview; Invalid = invalid preview.

Target word measures analyzed included the following: (a) first fixation duration (FFD; the initial first-pass fixation duration, regardless of whether the target was refixated); (b) single fixation duration (SFD; first-pass fixation time when a target was only fixated once); (c) gaze duration (GD; the sum of all first-pass fixations before the eyes move to another word); (d) total fixation time (TT; the sum of all fixations, including regressions and second-pass fixations); and skipping probability (PrSkip; the probability of skipping the target on its first-pass, calculated as a proportion of remaining trials after data loss). The raw distributions of fixation duration measures are illustrated by experimental design in Figure 1.

### Predictor coding and model choice

**Figure 2.**
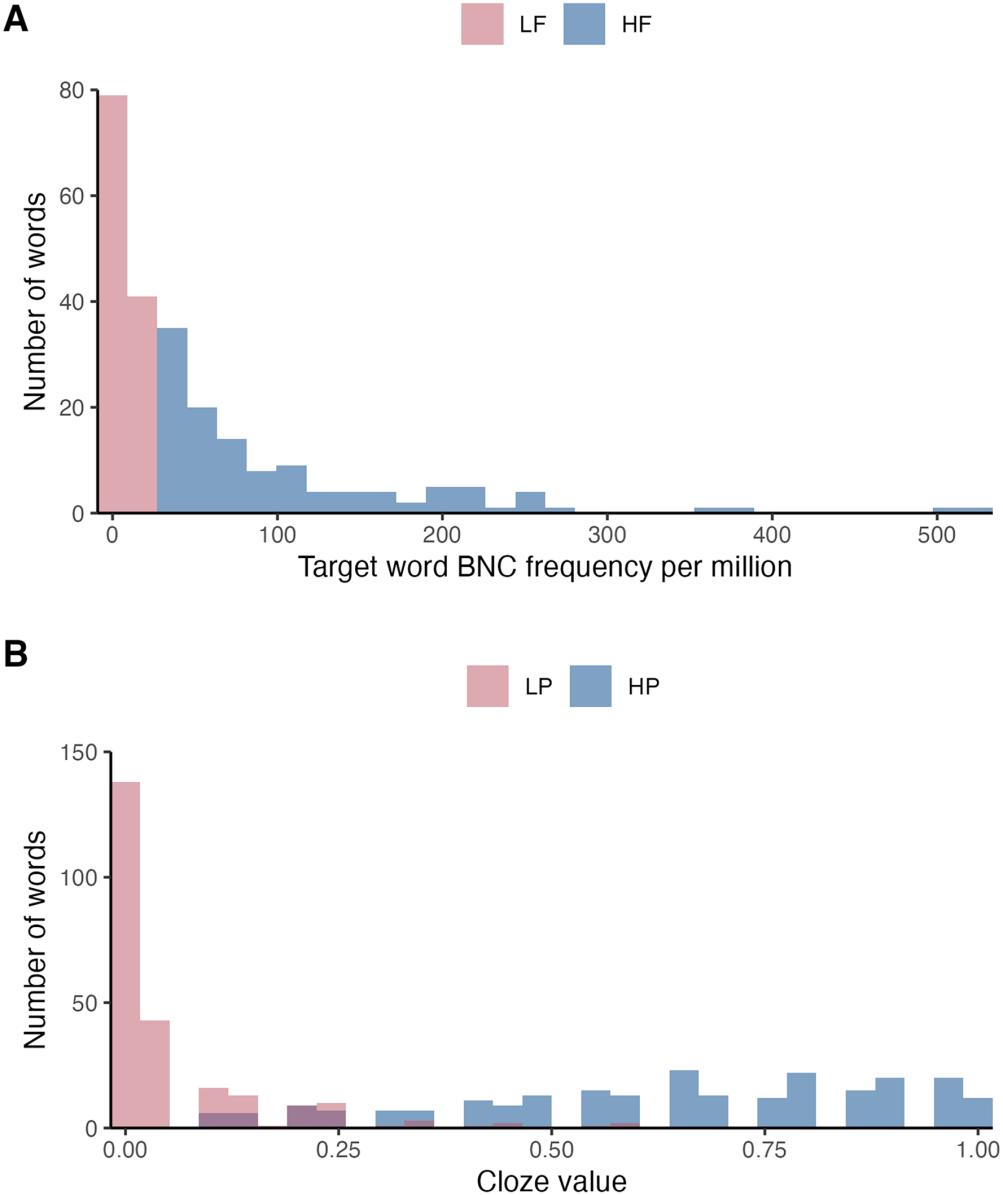
Histograms of target frequency and predictability values. (**A**) Frequency in occurrences per million written words; (**B**) Predictability in Cloze probability values.

Although the materials were designed categorically, with equal numbers of HF and LF words, and HP and LP words, we operationalized Frequency using the log-transformed British National Corpus written word frequency per million and Predictability by means of Cloze values of targets within biasing versus neutral contexts. Figure 2 shows that while the (raw) Frequency distributions were non-overlapping, the Predictability distributions did show some overlap. Therefore, to more precisely model the inter-word variations, we coded both (log) Frequency and Predictability as continuous variables in our models.

We used a Bayesian mixed-effects modeling (BMM) approach for several reasons: (1) to simultaneously model by-participant and by-target-word random effects within a single model; (2) to effectively model the positively skewed fixation time data using an ex-Gaussian distribution, which explicitly separates experimental effects on the distribution's center from its skew, providing fine-grained insights into how experimental manipulations differentially impact these parameters (Reingold et al., 2012; Staub, 2011; White & Staub, 2012; Yao et al., 2025); and (3) to appropriately model the binary PrSkip data using a Bernoulli distribution with a logit link (Collett, 2002). Moreover, this Bayesian approach uses Bayes factors to quantify the relative evidence strengths between H_1_ and H_0_, based on empirically informed prior distributions.

### Software packages

Data were analyzed using the *brms* package (Bürkner, 2017) in R (http://www.r-project.org). BMMs were fit using the brm() function. The Bayes factor (*BF_10_*) for fixed effects were computed using the hypothesis() function. Marginal conditional means and effect well calculated using the brmsmargins() function from the *brmsmargins* package (Wiley & Hedeker, 2022). Figures were generated using the *ggplot2* package (Wickham, 2016).

### BMMs

#### Specifying prior distributions

We specified empirically informed normal priors for fixed effects in our Bayesian mixed models. For the ex-Gaussian models of fixation durations, the prior for the intercept of *mu* was centered at 225 ms (*SD* = 50 ms), based on typical eye fixation times in reading research (Rayner, 1998). For the remaining intercepts, we used parameters reported by Staub (2011): setting the prior for *sigma* at 25 ms (*SD* = 5 ms) and the log-scale *beta* at 4.09 (equivalent to 60 ms) with an *SD* of 0.22 (approximating 12–15 ms variation around 60 ms).

For the Bernoulli models analyzing PrSkip data (on the log scale), the intercept prior was centered at −1.39, corresponding to the typical base skipping rate of around 0.25 observed in reading research (e.g., Rayner, 1998; Sereno et al., 2018). The *SD* was set to 0.5, which accommodates plausible variation in base skipping rates (between 0.08 and 0.29) 95% of the time.

In line with Dienes’ (2021) recommendations, we calibrated all fixed effect priors using empirically observed, representative effect sizes. In the ex-Gaussian fixation duration models, the prior for *mu* was specified using normal distributions centered at zero with an *SD* of 15 ms, derived from reported Frequency and Predictability effects (Staub, White, Drieghe, Hollway, & Rayner, 2010; Staub, 2015). Similarly, log-scale *beta* priors were normal distributions centered at zero with an *SD* of 0.24, reflecting word frequency effects on skew (∼10–15 ms on the raw scale) as reported by Staub et al. (2010). We specified variance priors for both participant and target word random effects using Student's *t*-distributions centered on zero, with 3 degrees of freedom and *SD*s matching those of the corresponding fixed effects.

For the Bernoulli skipping model, the prior for the fixed slope was specified using a normal distribution centered at zero with a *SD* of 0.3. This corresponds to approximately a 5% change in PrSkip around a baseline of 0.25, based on reported predictability effects on skipping (Rayner, 1998; Sereno et al., 2018).

Our prior specifications embodied our theoretical expectations about the fixed effects (both main effects and interactions). While centered at zero, these priors accommodated non-zero effects in both positive and negative directions, reflecting the uncertainty in how Frequency, Predictability and Preview may interact. The probability density favored effect sizes smaller than those typically reported in the literature, while allowing larger values.

This empirically informed approach to prior specification offers several advantages. Rather than arbitrarily defining a minimum effect size of interest, it leverages existing research to establish the range of plausible effects. Additionally, by centering priors at zero while allowing for non-zero effects, the method maintains a conservative stance toward hypothesis testing.

To test our hypotheses, we calculated Bayes factors (*BF_10_*) using the Savage-Dickey density ratio (Wagenmakers, Lodewyckx, Kuriyal, & Grasman, 2010). This method compares two models: one where the parameter of interest allows non-zero values (H_1_, based on our empirically informed priors), and another where the parameter is fixed at zero (H_0_). The resulting *BF_10_* quantifies the relative evidence for H_1_ over H_0_. This approach requires substantial evidence to favor either hypothesis, thereby balancing sensitivity to genuine effects with robust protection against false-positive results.

#### Prior predictive checks

We examined the distributional properties of prior samples from our BMMs to assess the plausibility of prior predictions for fixation durations. Simulated fixation durations and skipping probabilities exhibited positively skewed quasi-Gaussian distributions. Median fixation durations were around 225 ms (range, ∼100–400 ms), whereas median skipping probabilities were around 0.23 (range, 0.05–0.50). Scaled median absolute deviations ranged from approximately 30 to 125 ms for fixation durations and from approximately 0.05 to 0.45 for skipping probabilities (Figure C1 in Supplementary Materials C). The prior parameter estimates for fixed effects aligned with our prior specifications (Table C1 in Supplementary Materials C). These checks confirm that our prior specifications produce realistic fixation duration patterns and appropriate parameter ranges, strongly validating our modeling approach.

#### Model fitting

Fixation durations were modeled in ex-Gaussian BMMs with identity links for *mu* (central tendency) and *sigma* (dispersion), and a log link for *beta* (skewness). Skipping was modeled in a Bernoulli BMM with a logit link. We included three predictors: Frequency (between-word; continuous item-specific log(frequency) values), Predictability (within-word; continuous item-specific Cloze values), and Preview (within-word; binary). All predictors were centered and rescaled (*M* = 0, *SD* = 0.5). Fixed effects included all main effects and their interactions between Frequency, Predictability, and Preview. BMMs used maximal random effect structures with by-participant random intercepts and slopes for all effects, plus by-target-word random intercepts and slopes for within-word manipulations (Predictability and Preview). Models were fit with four chains of 5000 iterations (2500 warm-up), and all converged.

Table 2 reports coefficients with 95% Credible Intervals (CrIs) and *BF_10_*s. CrIs excluding zero indicate strong evidence for non-zero effects. *BF_10_* values quantify evidence strength for the alternative hypothesis versus the null, with *BF_10_* > 3 indicating moderate evidence (Jeffreys, 1998).

**Table 2.** BMM results by fixed effects and fixation measures.

Factor	*b(mu)*	*CrI_2.5_*	*CrI_97.5_*	*BF_10_*	*b(beta)*	*CrI_2.5_*	*CrI_97.5_*	*BF_10_*
Intercept								
FFD	227.64	221.71	233.66		4.08	4.01	4.16	
SFD	231.14	224.59	237.66		4.04	3.96	4.11	
GD	245.40	237.56	253.46		4.26	4.18	4.34	
TT	277.54	267.54	287.91		4.68	4.60	4.75	
PrSkip	−1.38	−1.56	−1.19					
Frequency								
FFD	**−4.10**	**−7.02**	**−1.15**	**3.88**	**−0.10**	**−0.15**	**−0.05**	**142.76**
SFD	**−4.54**	**−7.71**	**−1.43**	**5.17**	**−0.13**	**−0.19**	**−0.07**	**186.70**
GD	**−6.99**	**−10.56**	**−3.48**	**85.86**	**−0.12**	**−0.17**	**−0.07**	**152.08**
TT	**−6.99**	**−11.04**	**−2.88**	**30.56**	**−0.07**	**−0.11**	**−0.03**	**106.22**
PrSkip	0.08	−0.04	0.21	0.50				
Predictability								
FFD	**−5.66**	**−8.16**	**−3.13**	**>1000**	−0.06	−0.11	0	1.01
SFD	**−6.49**	**−9.34**	**−3.70**	**958.39**	*−0.06*	*−0.12*	*−0.01*	*1.37*
GD	**−7.05**	**−10.04**	**−4.14**	**>1000**	**−0.08**	**−0.13**	**−0.03**	**16.51**
TT	**−15.09**	**−19.06**	**−11.19**	**>1000**	**−0.11**	**−0.15**	**−0.07**	**>1000**
PrSkip	*0.10*	*0.02*	*0.19*	*2.12*				
Preview								
FFD	**−39.46**	**−44.40**	**−34.58**	**>1000**	**−0.41**	**−0.47**	**−0.34**	**>1000**
SFD	**−45.06**	**−50.79**	**−39.19**	**>1000**	**−0.38**	**−0.45**	**−0.30**	**>1000**
GD	**−54.44**	**−61.45**	**−47.29**	**>1000**	**−0.44**	**−0.51**	**−0.37**	**>1000**
TT	**−64.49**	**−73.37**	**−55.14**	**>1000**	**−0.38**	**−0.44**	**−0.32**	**>1000**
PrSkip	**0.84**	**0.70**	**0.97**	**>1000**				
Frequency × Predictability								
FFD	0.10	−4.80	5.19	0.17	0.04	−0.05	0.14	0.30
SFD	−0.09	−5.78	5.66	0.20	0.03	−0.08	0.14	0.28
GD	1.80	−3.71	7.46	0.23	0.07	−0.03	0.17	0.52
TT	4.13	−3.41	11.72	0.45	0.06	−0.02	0.13	0.43
PrSkip	−0.08	−0.25	0.09	0.44				
Frequency × Preview								
FFD	*6.32*	*0.84*	*11.68*	*2.29*	0.07	−0.02	0.16	0.62
SFD	*6.42*	*0.60*	*12.18*	*2.11*	0.06	−0.05	0.16	0.42
GD	**9.78**	**3.68**	**15.83**	**23.32**	0.08	−0.01	0.16	1.00
TT	**8.65**	**1.67**	**15.63**	**4.37**	0.02	−0.05	0.09	0.17
PrSkip	0.03	−0.15	0.22	0.34				
Predictability × Preview								
FFD	*−5.16*	*−10.10*	*−0.17*	*1.34*	0	−0.09	0.10	0.20
SFD	−4.00	−9.30	1.24	0.52	0.03	−0.08	0.14	0.27
GD	**−8.20**	**−13.72**	**−2.78**	**9.40**	−0.03	−0.12	0.06	0.23
TT	−2.54	−9.37	4.05	0.28	−0.03	−0.11	0.04	0.22
PrSkip	**0.21**	**0.04**	**0.38**	**4.86**				
Frequency × Predictability × Preview								
FFD	6.42	−3.14	15.80	0.76	0.14	−0.04	0.32	1.01
SFD	2.28	−7.38	12.19	0.36	0.08	−0.12	0.29	0.59
GD	7.42	−2.80	17.84	0.92	0.14	−0.04	0.31	1.12
TT	0.32	−12.31	12.91	0.41	0.05	−0.10	0.19	0.36
PrSkip	−0.02	−0.33	0.30	0.56				

*Note:* Fixed effects on *mu* (central tendency) and on *beta* (skew) are presented in the left and right sets of columns, respectively. Effects with CrIs not including zero and a *BF_10_* of more than 3 are highlighted in **bold**. Effects with CrIs not including zero or a *BF_10_* of more than 3, but not both, are *italicized*.

#### Posterior predictive checks

To validate our models’ ability to capture empirical patterns in the data, we extracted posterior samples, using posterior_predict(), and compared their distributions against observed fixation durations and skipping probabilities. Posterior predictions showed excellent correspondence with the observed data distributions across all measures (Figure C2 in Supplementary Materials C), with highly overlapping density and bar plots demonstrating that our models successfully capture both the central tendencies and the distributional characteristics of the empirical data. This close alignment between model predictions and observed patterns provides strong evidence for the adequacy of our BMMs in capturing the empirical characteristics of the observed data.

#### Model results

Marginal means and contrasts (in ms for fixation durations and in probabilities for skipping) were computed between the high (*M* + *SD*) and low (*M* – *SD*) levels of relevant factors, with their 95% CrIs reported in square brackets.

Our analyses revealed moderate to very strong evidence for the main effects of Frequency, Predictability, and Preview on both the *mu* and *beta* across almost all fixation measures, except for inconclusive main effects of Predictability on the *beta* for FFD and SFD. In line with the established literature, shorter fixation durations were observed for higher versus lower frequency targets, for higher versus lower predictability targets, and for targets having valid versus invalid previews. However, strong evidence emerged only for the main effect of Preview on PrSkip, showing increased skipping for valid versus invalid previews. Evidence for the main effect of Predictability on PrSkip remained inconclusive, though numerically greater skipping probability was observed for higher versus lower predictability targets (Table 2).

There was moderate to strong evidence for a Frequency × Preview interaction in GD and TT (*BF_10_*s > 4.37), but only weak evidence in FFD and SFD (with *BF_10_*s of 2.29 and 2.11, respectively). When unpacking the interaction, we observed a non-zero Frequency effect (HF-LF) for targets with Invalid previews (∆HF-LF_(FFD, SFD, GD, TT)_ = −7 [−12, −3], −8 [−12, −3], −12 [−17, −7], −11 [−17, −6], respectively), but the effect was not different from zero for targets with Valid previews (∆HF-LF_(FFD, SFD, GD, TT)_ = −1 [−5, 2], −1 [−5, 3], −2 [−6, 2], −3 [−7, 2], respectively).

There was moderate evidence for a Predictability × Preview interaction in GD (*BF_10_* = 9.40) and PrSkip (*BF_10_* = 4.86), and weak evidence in FFD (*BF_10_* = 1.34). The interaction pattern differed across measures. For FFD, GD, and PrSkip, Predictability effects emerged only with Valid previews (∆HP-LP_(FFD, GD, PrSkip)_ = −8 [−11, −5], −11 [−15, −8], 0.041 [0.019, 0.064]), but were not distinguishable from zero for targets with Invalid previews (∆HP-LP_(FFD, GD, PrSkip)_ = −3 [−7, 1], −3 [−7, 2], 0 [−0.016, 0.016]). In contrast, for SFD and TT, Predictability effects emerged under both preview conditions but were stronger with Valid previews (∆HP-LP_(SFD, TT)_ = −8 [−12, −5], −16 [−21, −12]) than with Invalid previews (∆HP-LP_(SFD, TT)_ = −4 [−9, 0], −14 [−19, −8]).

**Figure 3.**
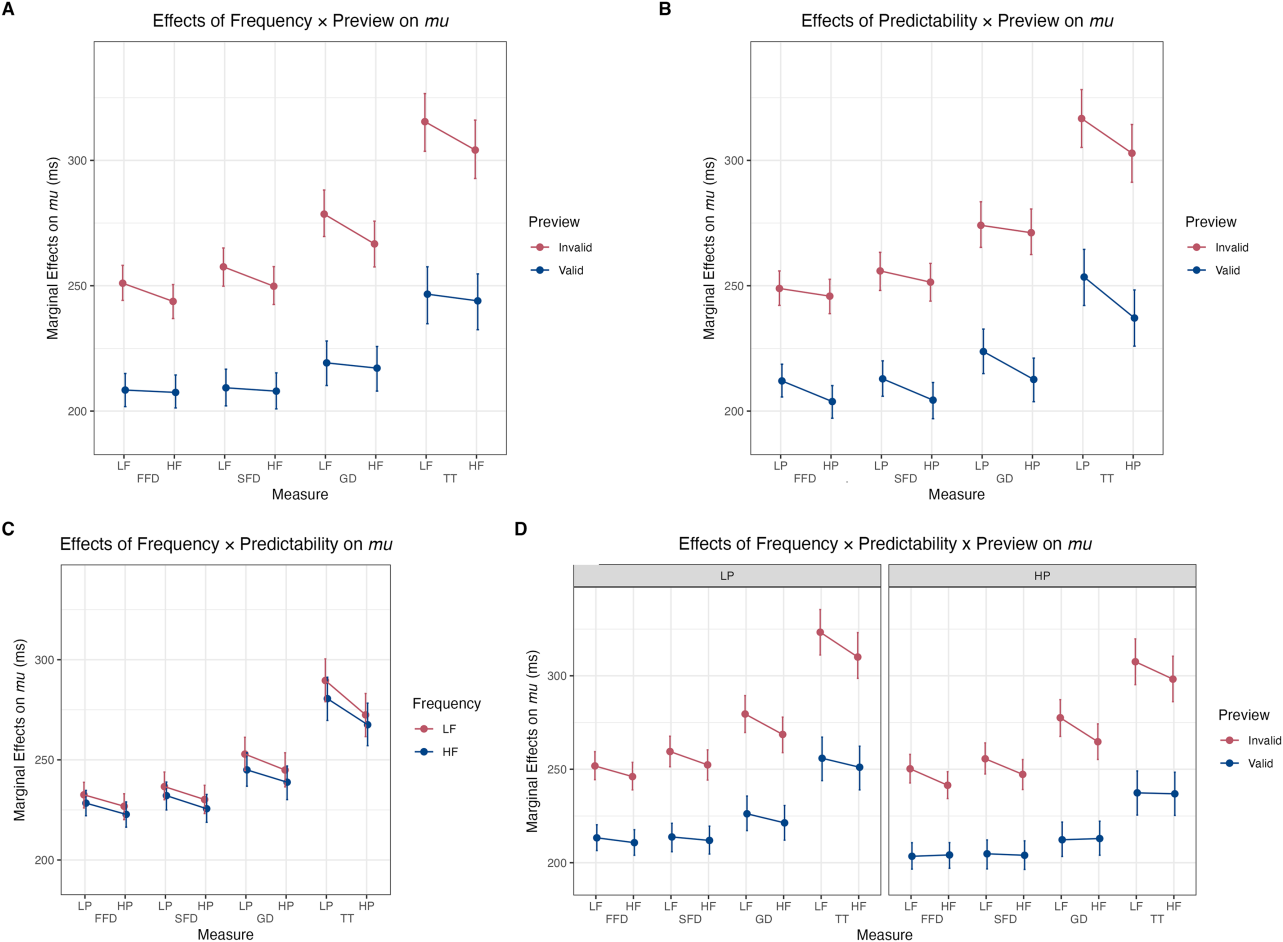
Interaction plots of effects across fixation duration measures. Interaction plots of (**A**) Frequency × Preview, (**B**) Predictability × Preview, (**C**) Frequency × Predictability, and (**D**) Frequency × Predictability × Preview. HF/LF and HP/LP are ± 1 *SD* from the mean of the log frequency and Cloze value, respectively. Valid = valid preview; Invalid = invalid preview.

**Figure 4.**
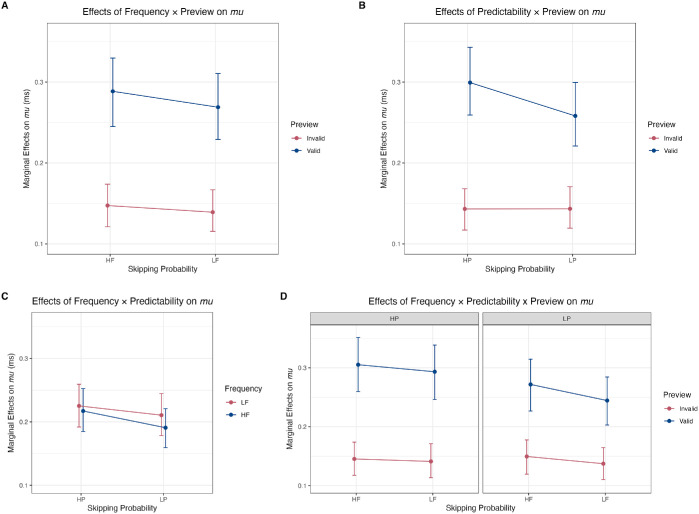
Interaction plots of effects on skipping probability. Interaction plots of (**A**) Frequency × Preview, (**B**) Predictability × Preview, (**C**) Frequency × Predictability, and (**D**) Frequency × Predictability × Preview. HF/LF and HP/LP are ± 1 *SD* from the mean of the log frequency and Cloze value, respectively. Valid = valid preview; Invalid = invalid preview.

There was no evidence for a three-way interaction between Frequency, Predictability, and Preview in any of the measures (with *BF_10_*s between 0.36 and 1.12). Plots of the significant interactions of Frequency × Preview (for GD and TT only) and Predictability × Preview (for GD and PrSkip only), as well as the non-significant Frequency × Predictability and three-way interactions, on the *mu* are shown in Figure 3 for fixation duration measures and Figure 4 for skipping probability.

Finally, to assess whether these effects persisted when accounting for variation in eye movement launch site (i.e., the eccentricity of the pre-target fixation), we conducted an additional analysis including launch distance as a covariate (Supplementary Materials D). The same pattern of Frequency × Preview and Predictability × Preview interactions for GD remained when controlling for launch distance, confirming that these effects are not attributable to lower-level oculomotor factors.

#### Sensitivity analysis

To examine how prior width specifications influence *BF_10_* values, we conducted a sensitivity analysis focusing on the most critical Frequency × Preview and Predictability × Preview interactions for the *mu* parameter. We systematically varied the prior scaling factor for the fixed effects of fixation durations, varying it from our original value of 15 ms to both a more conservative 10 ms and a more liberal 20 ms. For the fixed effects of PrSkip, we varied its scaling factor from 0.3 to 0.2 and 0.4 on a log scale, corresponding to probabilities of around 0.05, 0.03, and 0.07, respectively. The models were then refit with these alternative prior specifications, and *BF_10_*s were recalculated for the target interactions.

The results, shown in Table 3, demonstrated that *BF_10_*s for the key interactions—specifically the Frequency × Preview interactions in GD and TT, as well as the Predictability × Preview interaction in GD and skipping—remained stable across the range of prior widths tested. This stability provides confidence that our findings are robust to variations in prior specification.

**Table 3.** Effect sizes and Bayes factors for the Frequency × Preview and Predictability × Preview Interactions on the *mu* across various prior widths.

Prior Width: Fixation Duration	10		15		20	
Prior Width: Skipping Probability	0.2		0.3		0.4	
Factor	*mu*	*BF* *_10_*	*mu*	*BF* *_10_*	*mu*	*BF* *_10_*
Frequency × Preview						
FFD	*5.97*	*2.76*	*6.32*	*2.29*	*6.36*	*1.81*
SFD	*5.78*	*2.00*	*6.42*	*2.11*	*6.39*	*1.71*
GD	**9.07**	**21.05**	**9.78**	**23.32**	**9.98**	**20.06**
TT	**8.65**	**4.37**	**8.65**	**4.37**	**8.94**	**3.73**
PrSkip	0.03	0.50	0.03	0.34	0.03	0.26
Predictability × Preview						
FFD	*−5.03*	*1.79*	*−5.16*	*1.34*	*−5.25*	*0.94*
SFD	−4.01	0.87	−4.00	0.52	−4.19	0.47
GD	−**7.83**	**12.62**	**−8.20**	**9.40**	**−8.27**	**10.26**
TT	−2.54	0.28	−2.54	0.28	−2.50	0.22
PrSkip	**0.19**	**6.28**	**0.21**	**4.86**	**0.22**	**4.16**

*Note*: Effects with CrIs not including zero and a *BF_10_* of more than 3 are highlighted in **bold**. Effects with CrIs not including zero or a *BF_10_* of more than 3, but not both, are *italicized*.

## Discussion

This study investigated how parafoveal preview modulates the interaction between word frequency and contextual predictability during reading. Using a within-participants design and tightly controlled, item-matched stimuli, we used Bayesian ex-Gaussian mixed-effects models to analyze fixation durations and Bayesian Bernoulli mixed models for skipping probabilities on target words. Our goal was to clarify the nature of frequency and predictability effects under different preview conditions, building on and extending the findings of Sereno et al. (2018) and Staub and Goddard (2019). Our results replicated the established main effects of word frequency, contextual predictability, and preview validity. More critically, we observed specific two-way interactions involving parafoveal preview on the *mu* of fixation durations, namely a Frequency × Preview interaction evident primarily in GD and TT, and a Predictability × Preview interaction on the *mu* of GD and skipping probabilities. We found no evidence for a Frequency × Predictability interaction, nor for the three-way Frequency × Predictability × Preview interaction.

The nature of how frequency and predictability effects are modulated by parafoveal preview offers insights into word processing in reading. The observed Frequency × Preview interaction in GD and TT indicated that word frequency effects were most pronounced with invalid previews, and were significantly reduced with valid previews. This pattern suggests that when parafoveal preview is invalid but maintains target-like visual characteristics (as with our shape-matched pseudowords), readers must override misleading parafoveal information during foveal processing, and this corrective process appears to be facilitated by higher word frequency. Conversely, when preview is valid, the congruent foveal information eliminates the need for corrective processing, thereby reducing frequency-based advantages.

Notably, this finding contrasts with Reingold et al.’s (2012) observation of greater frequency effects with valid preview. This discrepancy may reflect critical differences in invalid preview characteristics: while our study employed shape-matching pseudowords that preserved targets’ visual contours, Reingold et al. used shape-dissimilar pronounceable pseudowords. Shape-dissimilar invalid previews may be sufficiently disruptive to prevent meaningful parafoveal processing entirely, creating conditions wherein valid previews provide maximal benefit and frequency effects are strongest. In contrast, shape-matched invalid previews may permit partial parafoveal processing of visual features while concurrently providing misleading lexical information, triggering frequency-dependent override mechanisms during subsequent foveal processing. This suggests that the specific visual and orthographic properties of invalid previews critically determine how frequency effects manifest across preview conditions. The emergence of this interaction primarily in GD suggests that these frequency-dependent override mechanisms unfold during sustained word processing rather than the initial lexical access stages.

Moreover, a Predictability × Preview interaction was found primarily in GD and skipping, where the facilitatory effect of higher predictability (HP vs. LP) was most pronounced when a valid preview was available, a pattern similar to that found in both Sereno et al. (2018) and Staub and Goddard (2019). This suggests that contextual predictability is most beneficial when parafoveal information supports the integration of top-down expectations with bottom-up visual input. The appearance of this interaction in skipping, a pre-foveal measure, indicates that this integration process begins even before the word is directly fixated. For words that are not skipped, its re-emergence in GD suggests that the process culminates during the first-pass foveal fixation. The lack of a clear effect on FFD implies that the benefit of this integration does not necessarily speed up early visual and lexical processing, but primarily reduces the likelihood that a second fixation is needed, thereby shortening GD. Conversely, when preview is invalid, the misleading parafoveal input may obfuscate the early integration of predictive context until the word is directly fixated.

Notably, these preview interactions emerged in the *mu* parameter but not the *beta* parameter of our ex-Gaussian models, indicating that parafoveal information shifts the entire distribution of fixation times uniformly rather than disproportionately affecting only the most “challenging words”—for example, those with lower predictability, frequency, familiarity, arousal, and dominance values, and later age of acquisition (Yao et al., 2025). This suggests that preview quality influences core reading mechanisms systematically across all lexical encounters.

An additional consideration concerns the relative strengths of frequency and predictability effects in our data, and how these compare to findings from recent large-scale re-analyses. In the present data, predictability main effects were overall stronger than frequency main effects, consistent with a large body of evidence that contextual constraint exerts a powerful influence on eye movement behavior overall during reading. This finding converges with recent work by Chandra, Witzig, and Laubrock (2023), who applied large language model-derived “synthetic predictabilities” to re-analyze multiple existing datasets and likewise found predictability to be the dominant linguistic predictor of fixation durations across studies. This dominance is further reflected in predictability effects being less influenced by preview than frequency effects, suggesting that predictability's role in contextual integration is robust and stable, while lexical access is more sensitive to preview conditions.

In contrast, the absence of a Frequency × Predictability interaction across our measures is noteworthy. While some eye movement studies have reported such interactions with “ultra-high” predictability (e.g., Sereno et al., 2018, with valid previews), our findings suggest that, under the current experimental conditions (i.e., in terms of Cloze value, our HP condition was more comparable to Sereno et al.’s (2018) MP than their HP condition) and with our analytical approach, these two factors did not significantly modulate each other. Similarly, the lack of a three-way Frequency × Predictability × Preview interaction contrasts with some previous findings (e.g., Sereno et al., 2018, reported a three-way interaction for early fixation time measures with valid previews). The lack of a Frequency × Predictability interaction is striking given that both factors independently interacted with Preview in GD.

The temporal dissociation between frequency and predictability processing, as revealed by electrophysiological research, may explain this independence despite their concurrent influence on GD. In an event-related potential frequency-predictability reading study, Sereno et al. (2019) demonstrated that predictability effects emerged earlier (50-80 ms post-stimulus) than frequency effects (80-120 ms), though both were sustained through later processing stages including the N400 component (350-550 ms). This temporal offset suggests that although both factors contribute to GD—a measure that encompasses the cumulative processing time across multiple processing stages—they may reflect fundamentally different computational processes that unfold in parallel rather than interactively.

Specifically, frequency effects may primarily reflect the efficiency of lexical access mechanisms, with higher frequency words benefiting from more robust and accessible and representations within the mental lexicon. In contrast, predictability effects predominantly involve contextual integration processes, where preceding semantic context facilitates word recognition through top-down constraint satisfaction. The findings that both Frequency and Predictability interactions with Preview emerged in GD suggests that this measure captures a processing window during which both lexical access and contextual integration processes are active, but their separable neural time courses may preclude direct interaction between these mechanisms.

Furthermore, the differential preview requirements for these effects—with frequency effects emerging only under invalid preview conditions while predictability effects require valid preview for optimal expression—support the notion that these factors engage distinct processing pathways. Frequency-based processing appears to be a compensatory mechanism activated when parafoveal information is unreliable, whilst predictability-based processing depends on the successful integration of contextual expectations with coherent parafoveal input. This functional dissociation aligns with Kretzschmar et al.’s (2015) findings of additive rather than interactive effects of frequency and predictability in both eye movement and EEG measures, reinforcing the independence of these processing streams even when both contribute to reading fluency. Moreover, the stability of our findings when launch distance (i.e., pre-target eccentricity) was included as a covariate confirms that cognitive rather than oculomotor influences are at play.

Comparing our findings with previous key studies highlights several important points. Sereno et al. (2018), who also used shape-matched pseudoword invalid previews, found a Predictability × Preview interaction in their between-experiment analysis: with valid preview, both MP and (ultra) HP targets were facilitated relative to LP targets; with invalid preview, only (ultra) HP targets were facilitated. Our Predictability × Preview interaction in GD (greater predictability effect with valid preview) is broadly consistent with the idea that invalid preview impedes the utility of predictability. Staub and Goddard (2019) used shape-matched semantically anomalous words or consonant strings as invalid previews and found that the predictability effect disappeared with invalid previews, consistent with our Predictability × Preview interaction. However, our Frequency × Preview interaction (larger frequency effect with invalid preview in GD) differs from both Sereno et al. (2018) and Staub and Goddard (2019) who found no such interaction. This may stem from multiple factors, including the precise nature and potential disruptiveness of the invalid previews (e.g., pseudowords, semantically anomalous words, vs. consonant strings), differences in the operationalization and range of target specifications (e.g., word length, frequency, and predictability levels), design choices (e.g., within- vs. between-participant preview manipulation; target repetition), and the statistical modeling approaches used (e.g., analyses of variance vs. Bayesian mixed-effects models with continuous predictors).

The principal strengths of our study are related to its design and materials. LF and HF target words were presented in two-sentence passages, in which the first sentence established a biasing or neutral context. The target was embedded in the second sentence which was identical across predictability conditions. In addition, the pre-target region of the second sentence was relatively neutral and did not contain, for example, intralexical (i.e., associative or semantic) primes of the subsequent target. In comparison to past eye movement research, our study is unique in its manipulation of frequency, predictability, and preview all within a repeated-measures design, notably without the repetition of target words and, at the same time, maintaining identical local contexts across predictability conditions. In addition, the materials, themselves, represent a considerably large set of well-controlled items. Moreover, the item-specific condition values were effectively exploited via Bayesian mixed-effects models for analysis, allowing for a nuanced examination of distributional properties of fixation times.

One limitation of the current study is that targets were restricted to four- and five-letter words. Although target word length influences the saccade length into that word as well as the fixation location itself, its effects have been shown to be independent of frequency and predictability (e.g., Juhasz, White, Liversedge, & Rayner, 2008; Rayner, 1979; Rayner et al., 1996; Rayner, Slattery, Drieghe, & Liversedge, 2011). Nevertheless, the use of longer targets (that still fall within the word identification span; e.g., Rayner, 1998) would enhance the generalizability of the current findings.

Another limitation of our study is that the Cloze values for our higher predictability targets were somewhat variable. While having a well-populated range of predictability values is advantageous for robust statistics, particularly when treating predictability as a continuous variable, there is evidence demonstrating a categorically different pattern of effects, however, when predictability levels are very high, for example, with targets having Cloze values greater than 0.90 (e.g., Sereno et al., 2018). Such ultra-high levels of predictability should continue to be tested more rigorously in future research.

Future work could also make greater use of alternative approaches for assessing the role of parafoveal preview—such as visual degradation of the target word (Gagl et al., 2014), launch site analyses (Hand et al., 2010), or parafoveal magnification (Miellet et al., 2009; Yao et al., 2025)—which vary the extent of parafoveal information without introducing an erroneous stimulus.

## Conclusions

Our findings reveal that parafoveal preview quality modulates how word frequency and contextual predictability influence lexical processing during reading. The emergence of a Frequency × Preview interaction, where frequency effects were greater under invalid preview conditions, suggests that high frequency words provide a processing advantage specifically when readers must override misleading parafoveal information. Conversely, the Predictability × Preview interaction demonstrates that contextual facilitation depends critically on coherent parafoveal input to support the integration of top-down expectations with bottom-up visual processing.

In contrast, the absence of a Frequency × Predictability interaction, despite both factors independently interacting with preview conditions, supports a model of lexical processing in which frequency-based lexical access and predictability-based contextual integration operate through distinct, parallel pathways rather than interactive mechanisms. This functional dissociation suggests they reflect separable computational processes with different temporal dynamics and preview requirements.

These results advance our understanding of how parafoveal information shapes word recognition in reading, demonstrating that the quality and congruence of preview information determine not just the magnitude of processing effects, but the very nature of how frequency and predictability influence reading. This has important implications for models of eye movement control and lexical processing, highlighting the need to consider parafoveal preview characteristics when interpreting frequency and predictability effects in reading research.

## Supplementary Material

Supplement 1

Supplement 2

Supplement 3

Supplement 4

## References

[bib1] Angele, B., Slattery, T. J., & Rayner, K. (2016). Two stages of parafoveal processing during reading: Evidence from a display change detection task. *Psychonomic Bulletin and Review,* 23(4), 1241–1249, 10.3758/s13423-015-0995-0. 26769246 PMC4974265

[bib2] Anstis, S. M. (1974). A chart demonstrating variations in acuity with retinal position. *Vision Research,* 14(7), 589–592, 10.1016/0042-6989(74)90049-2. 4419807

[bib3] Balota, D. A., Pollatsek, A., & Rayner, K. (1985). The interaction of contextual constraints and parafoveal visual information in reading. *Cognitive Psychology,* 17(3), 364–390, 10.1016/0010-0285(85)90013-1. 4053565

[bib4] Balota, D. A., & Rayner, K. (1991). Word recognition processes in foveal and parafoveal vision: The range of influence of lexical variables. In D. Besner & G. W. Humphreys (Eds.), *Basic processes in reading: Visual word recognition* (pp. 198–232). Mahwah, NJ: Lawrence Erlbaum Associates, Inc.

[bib7] Becker, W. (1989). Metrics. In R. H. Wurtz & M. E. Goldberg (Eds.), *The neurobiology of saccadic eye movements* (pp. 13–67). New York: Elsevier. 2486323

[bib8] Bürkner, P.-C. (2017). brms: An R package for Bayesian multilevel models using Stan. *Journal of Statistical Software,* 80, 1–28, 10.18637/jss.v080.i01.

[bib9] Brysbaert, M., & Stevens, M. (2018). Power analysis and effect size in mixed effects models: A tutorial. *Journal of Cognition,* 1(1), 9, 1–20, 10.5334/joc.10. PMC6646942 31517183

[bib10] Chandra, J., Witzig, N., & Laubrock, J. (2023). Synthetic predictabilities from large language models explain reading eye movements. *ERTS ‘23: Proceedings of the 2023 Symposium on Eye Tracking Research and Applications,* Article 19, 1–7, 10.1145/3588015.3588420.

[bib11] Collett, D. (2002). *Modelling binary data*. Boca Raton, FL: CRC Press, 10.1201/b16654.

[bib12] Dambacher, M., Dimigen, O., Braun, M., Wille, K., Jacobs, A. M., & Kliegl, R. (2012). Stimulus onset asynchrony and the timeline of word recognition: Event-related potentials during sentence reading. *Neuropsychologia,* 50(8), 1852–1870, 10.1016/j.neuropsychologia.2012.04.011. 22564485

[bib13] Dambacher, M., Kliegl, R., Hofmann, M., & Jacobs, A. M. (2006). Frequency and predictability effects on event-related potentials during reading. *Brain Research,* 1084(1), 89–103, 10.1016/j.brainres.2006.02.010. 16545344

[bib14] Davies, M. (2004). *British National Corpus*. https://www.english-corpora.org/bnc/.

[bib15] Dienes, Z. (2021). Obtaining evidence for no effect. *Collabra: Psychology,* 7(1), 28202, 10.1525/collabra.28202.

[bib16] Fodor, J. A. (1983). *Modularity of mind*. Cambridge, MA: MIT Press, 10.7551/mitpress/4737.001.0001.

[bib17d] Ehrlich, S. F., & Rayner, K. (1981). Contextual effects on word perception and eye movements during reading. *Journal of Verbal Learning and Verbal Behavior,* 20(6), 641–655, 10.1016/S0022-5371(81)90220-6.

[bib17] Gagl, B., Hawelka, S., Richlan, F., Schuster, S., & Hutzler, F. (2014). Parafoveal preprocessing in reading revisited: Evidence from a novel preview manipulation. *Journal of Experimental Psychology: Learning, Memory, and Cognition**,* 40(2), 588–595, 10.1037/a0034408. 24041397

[bib18] Hand, C. J., Miellet, S., O'Donnell, P. J., & Sereno, S. C. (2010). The frequency-predictability interaction in reading: It depends where you're coming from. *Journal of Experimental Psychology: Human Perception and Performance**,* 36(5), 1294–1313, 10.1037/a0020363. 20854004

[bib19] Hand, C. J., O'Donnell, P. J., & Sereno, S. C. (2012). Word-initial letters influence fixation durations during fluent reading. *Frontiers in Psychology: Language Sciences**,* 3*,* Article 85, 1–19, 10.3389/fpsyg.2012.00085. PMC3317262 22485100

[bib20] Ingram, J., & Hand, C. J. (2020). Words from the wizarding world: Fictional words, context, and domain knowledge. *Journal of Experimental Psychology: Learning, Memory, and Cognition,* 46(11), 2179–2192, 10.1037/xlm0000946. 32757581

[bib21] Inhoff, A. W. (1984). Two stages of word processing during eye fixations in the reading of prose. *Journal of Verbal Learning and Verbal Behavior,* 23(5), 612–624, 10.1016/S0022-5371(84)90382-7.

[bib22] Inhoff, A. W., & Rayner, K. (1986). Parafoveal word processing during eye fixations in reading: Effects of word frequency. *Perception & Psychophysics,* 40(6), 431–439, 10.3758/BF03208203. 3808910

[bib23] Jeffreys, H. (1998). *The theory of probability*. Oxford, UK: Oxford University Press.

[bib24] Johnson, R. L., Perea, M., & Rayner, K. (2007). Transposed-letter effects in reading: Evidence from eye movements and parafoveal preview. *Journal of Experimental Psychology: Human Perception and Performance,* 33(1), 209–229, 10.1037/0096-1523.33.1.209. 17311489

[bib25] Juhasz, B. J., White, S. J., Liversedge, S. P., & Rayner, K. (2008). Eye movements and the use of parafoveal word length information in reading. *Journal of Experimental Psychology: Human Perception and Performance,* 34(6), 1560–1579, 10.1037/a0012319. 19045993 PMC2668122

[bib26] Just, M. A., & Carpenter, P. A. (1980). A theory of reading: From eye fixations to comprehension. *Psychological Review,* 87(4), 329–354, 10.1037/0033-295X.87.4.329. 7413885

[bib27] Kennedy, A., Pynte, J., Murray, W. S., & Paul, S.-A. (2013). Frequency and predictability effects in the Dundee Corpus: An eye movement analysis. *Quarterly Journal of Experimental Psychology,* 66(3), 601–618, 10.1080/17470218.2012.676054. 22643118

[bib28] Kliegl, R., Grabner, E., Rolfs, M., & Engbert, R. (2004). Length, frequency, and predictability effects of words on eye movements in reading. *European Journal of Cognitive Psychology,* 16(1-2), 262–284, 10.1080/09541440340000213.

[bib29] Kowler, E. (2011). Eye movements: The past 25 years. *Vision Research,* 51(13), 1457–1483, 10.1016/j.visres.2010.12.014. 21237189 PMC3094591

[bib30] Kretzschmar, F., Schlesewsky, M., & Staub, A. (2015). Dissociating word frequency and predictability effects in reading: Evidence from coregistration of eye movements and EEG. *Journal of Experimental Psychology: Learning, Memory, and Cognition**,* 41(6), 1648–1662, 10.1037/xlm0000128. 26010829

[bib31] Kruschke, J. K., & Liddell, T. M. (2018). The Bayesian new statistics: Hypothesis testing, estimation, meta-analysis, and power analysis from a Bayesian perspective. *Psychonomic Bulletin & Review,* 25(1), 178–206, 10.3758/s13423-016-1221-4. 28176294

[bib32] Kveraga, K., Boshyan, J., & Bar, M. (2007). Magnocelllular projections as the trigger of top-down facilitation in recognition. *Journal of Neuroscience,* 27(48), 13232–13240, 10.1523/JNEUROSCI.3481-07.2007. 18045917 PMC6673387

[bib33] Levy, R. (2008). Expectation-based syntactic comprehension. *Cognition,* 106(3), 1126–1177, 10.1016/j.cognition.2007.05.0. 17662975

[bib34] Lima, S. D., & Inhoff, A. W. (1985). Lexical access during eye fixations in reading: Effects of word-initial letter sequence. *Journal of Experimental Psychology: Human Perception and Performance**,* 11(3), 272–285, 10.1037/0096-1523.11.3.272. 3159838

[bib35] Mayzner, M. S., & Tresselt, M. E. (1965). Tables of single-letter and diagram frequency for various word-length and letter-position combinations. *Psychonomic Monograph Supplements,* 1(2), 13–32.

[bib36] McClelland, J. L. (1987). The case for interactionism in language processing. In M. Coltheart (Ed.), *Attention and performance XII. The psychology of reading* (pp. 363–383). Mahwah, NJ: Lawrence Erlbaum, 10.4324/9781315630427.

[bib37] McConkie, G. W., & Rayner, K. (1975). The span of the effective stimulus during a fixation in reading. *Perception & Psychophysics**,* 17(6)*,* 578–586, 10.3758/BF03203972.

[bib38] McConkie, G. W., & Zola, D. (1979). Is visual information integrated across successive fixations in reading? *Perception & Psychophysics,* 25(3), 221–224, 10.3758/BF03202990. 461078

[bib39] McDonald, S. A., & Shillcock, R. C. (2003). Eye movements reveal the on-line computations of lexical probabilities during reading. *Psychological Science,* 14(6), 648–652, 10.1046/j.0956-7976.2003.psci_1480.x. 14629701

[bib40] Miellet, S., O'Donnell, P. J., & Sereno, S. C. (2009). Parafoveal magnification: Visual acuity does not modulate the perceptual span in reading. *Psychological Science,* 20(6), 721–728, 10.1111/j.1467-9280.2009.02364.x. 19470124

[bib41] Miellet, S., Sparrow, L., & Sereno, S. C. (2007). Word frequency and predictability effects in reading French: An evaluation of the E-Z Reader model. *Psychonomic Bulletin & Review,* 14(4), 762–769, 10.3758/BF03196834. 17972746

[bib42] Morris, R. K. (1994). Lexical and message level sentence context effects on fixation times in reading. *Journal of Experimental Psychology: Learning, Memory, and Cognition,* 20(1), 92–103, 10.1037/0278-7393.20.1.92. 8138791

[bib44] Penolazzi, B., Hauk, O., & Pulvermüller, F. (2007). Early semantic context integration and lexical access as revealed by event-related brain potentials. *Biological Psychology,* 74(3), 374–388, 10.1016/j.biopsycho.2006.09.008. 17150298

[bib45] Pollatsek, A., Lesch, M., Morris, R. K., & Rayner, K. (1992). Phonological codes are used in integrating information across saccades in word identification and reading. *Journal of Experimental Psychology: Human Perception and Performance**,* 18(1)*,* 148–162, 10.1037/0096-1523.18.1.148. 1532185

[bib46] Rayner, K. (1975). The perceptual span and peripheral cues in reading. *Cognitive Psychology,* 7(1), 65–81, 10.1016/0010-0285(75)90005-5.

[bib47] Rayner, K. (1979). Eye guidance in reading: Fixation locations within words. *Perception,* 8(1), 21–30, 10.1068/p080021. 432075

[bib48] Rayner, K. (1998). Eye movements in reading and information processing: 20 years of research. *Psychological Bulletin,* 124(3), 372–422, 10.1037/0033-2909.124.3.372. 9849112

[bib49] Rayner, K. (2009). The 35^th^ Sir Frederick Bartlett Lecture: Eye movements and attention in reading, scene perception, and visual search. *Quarterly Journal of Experimental Psychology,* 62(8), 1457–1506, 10.1080/17470210902816461. 19449261

[bib50] Rayner, K., Ashby, J., Pollatsek, A., & Reichle, E. D. (2004). The effects of frequency and predictability on eye fixations in reading: Implications for the E-Z Reader model. *Journal of Experimental Psychology: Human Perception and Performance,* 30(4), 720–730. 10.1037/0096-1523.30.4.720. 15301620

[bib51] Rayner, K., & Duffy, S. A. (1986). Lexical complexity and fixation times in reading: Effects of word frequency, verb complexity, and lexical ambiguity. *Memory & Cognition,* 14(3), 191–201, 10.3758/BF03197692. 3736392

[bib52] Rayner, K., Sereno, S. C., & Raney, G. E. (1996). Eye movement control in reading: A comparison of two types of models. *Journal of Experimental Psychology: Human Perception and Performance,* 22(5), 1188–1200, 10.1037/0096-1523.22.5.1188. 8865619

[bib53] Rayner, K., Slattery, T. J., Drieghe, D., & Liversedge, S. P. (2011). Eye movements and word skipping during reading: Effects of word length and predictability. *Journal of Experimental Psychology: Human Perception and Performance,* 37(2), 514–528, 10.1037/a0020990. 21463086 PMC3543826

[bib54] Rayner, K., & Well, A. D. (1996). Effects of contextual constraint on eye movements in reading: A further examination. *Psychonomic Bulletin & Review,* 3(4), 504–509, 10.3758/BF03214555. 24213985

[bib55] Rayner, K., Well, A. D., Pollatsek, A., & Bertera, J. H. (1982). The availability of useful information to the right of fixation in reading. *Perception & Psychophysics,* 31(6), 537–550, 10.3758/BF03204186. 7122189

[bib56] Reingold, E. M., Reichle, E. D., Glaholt, M. G., & Sheridan, H. (2012). Direct lexical control of eye movements in reading: Evidence from a survival analysis of fixation durations. *Cognitive Psychology,* 65(2), 177–206, 10.1016/j.cogpsych.2012.03.001. 22542804 PMC3565237

[bib57] Schotter, E. R., Angele, B., & Rayner, K. (2012). Parafoveal processing in reading. *Attention, Perception, & Psychophysics,* 74(1), 5–35, 10.3758/s13414-011-0219-2. 22042596

[bib58] Scott, G. G., O'Donnell, P. J., & Sereno, S. C. (2012). Emotion words affect eye fixations during reading. *Journal of Experimental Psychology: Learning, Memory, and Cognition,* 38(3), 783–792, 10.1037/a0027209. 22329788

[bib59] Sereno, S. C. (1992). Early lexical effects when fixating a word in reading. In K. Rayner (Ed.), *Eye movements and visual cognition: Scene perception and reading* (pp. 304–316). New York: Springer-Verlag.

[bib60] Sereno, S. C. (1995). Resolution of lexical ambiguity: Evidence from an eye movement priming paradigm. *Journal of Experimental Psychology: Learning, Memory, and Cognition,* 21(3), 582–595, 10.1037/0278-7393.21.3.582. 7602263

[bib61] Sereno, S. C., Brewer, C. C., & O'Donnell, P. J. (2003). Context effects in word recognition: Evidence for early interactive processing. *Psychological Science,* 14(4), 328–333, 10.1111/1467-9280.14471. 12807405

[bib62] Sereno, S. C., Hand, C. J., Shahid, A., Mackenzie, I. G., & Leuthold, H. (2019). Early EEG correlates of word frequency and contextual predictability in reading. *Language, Cognition and Neuroscience,* 35(5), 625–640, 10.1080/23273798.2019.1580753.

[bib63] Sereno, S. C., Hand, C. J., Shahid, A., Yao, B., & O'Donnell, P. J. (2018). Testing the limits of contextual constraint: Interactions with frequency and parafoveal preview during fluent reading. *Quarterly Journal of Experimental Psychology**,* 71(1), 302–313, 10.1080/17470218.2017.1327981. PMC6159772 28481189

[bib64d] Sereno, S. C., O'Donnell, P. J., & Rayner, K. (2006). Eye movements and lexical ambiguity resolution: Investigating the subordinate bias effect. *Journal of Experimental Psychology: Human Perception and Performance,* 32(2), 335–350, 10.1037/0096-1523.32.2.335. 16634674

[bib64] Sereno, S. C., Pacht, J. M., & Rayner, K. (1992). The effect of meaning frequency on processing lexically ambiguous words: Evidence from eye fixations. *Psychological Science,* 3(5), 296–300, 10.1111/j.1467-9280.1992.tb00676.x.

[bib65] Sereno, S. C., & Rayner, K. (1992). Fast priming during eye fixations in reading. *Journal of Experimental Psychology: Human Perception and Performance,* 18(1), 173–184, 10.1037/0096-1523.18.1.173. 1532187

[bib66] Sereno, S. C., & Rayner, K. (2000). Spelling-sound regularity effects on eye fixations in reading. *Perception & Psychophysics,* 62(2), 402–409, 10.3758/BF03205559. 10723218

[bib67] Slattery, T. J., Pollatsek, A., & Rayner, K. (2007). The effect of the frequencies of three consecutive content words on eye movements during reading. *Memory & Cognition,* 35(6), 1283–1292, 10.3758/BF03193601. 18035627

[bib68] Slattery, T. J., Staub, A., & Rayner, K. (2012). Saccade launch site as a predictor of fixation durations in reading: Comments on Hand, Miellet, O'Donnell, & Sereno (2010). *Journal of Experimental Psychology: Human Perception and Performance,* 38(1), 251–261, 10.1037/a0025980. 22082213

[bib69] Stanovich, K. E., & West, R. F. (1983). On priming by sentence context. *Journal of Experimental Psychology: General,* 112(1), 1–36, 10.1037/0096-3445.112.1.1. 6221061

[bib70] Staub, A. (2011). The effect of lexical predictability on distributions of eye fixation durations. *Psychonomic Bulletin & Review,* 18(2), 371–376, 10.3758/s13423-010-0046-9. 21327339

[bib71] Staub, A. (2015). The effect of lexical predictability on eye movements in reading: Critical review and theoretical interpretation. *Language and Linguistics Compass,* 9(8), 311–327, 10.1111/lnc3.12151.

[bib72] Staub, A., & Goddard, K. (2019). The role of preview validity in predictability and frequency effects on eye movements in reading. *Journal of Experimental Psychology: Learning, Memory, and Cognition,* 45(1), 110–127, 10.1037/xlm0000561. 29648870

[bib73d] Staub, A., White, S. J., Drieghe, D., Hollway, E. C., & Rayner, K. (2010). Distributional effects of word frequency on eye fixation durations. *Journal of Experimental Psychology: Human Perception and Performance,* 36(5), 1280–1293, 10.1037/a0016896. 20873939 PMC2948239

[bib74] van Petten, C., & Kutas, M. (1990). Interactions between sentence context and word frequency in event-related brain potentials. *Memory & Cognition,* 18(4), 380–393, 10.3758/BF03197127. 2381317

[bib75] Veldre, A., & Andrews, S. (2018). Parafoveal preview effects depend on both preview plausibility and target predictability. *Quarterly Journal of Experimental Psychology,* 71(1), 64–74, 10.1080/17470218.2016.1247894. 27734767

[bib76] Wagenmakers, E.-J., Lodewyckx, T., Kuriyal, H., & Grasman, R. (2010). Bayesian hypothesis testing for psychologists: A tutorial on the Savage-Dickey method. *Cognitive Psychology,* 60(3), 158–189, 10.1016/j.cogpsych.2009.12.001. 20064637

[bib77] West, R. F., & Stanovich, K. E. (1982). Source of inhibition in experiments on the effect of sentence context on word recognition. *Journal of Experimental Psychology: Learning, Memory, and Cognition,* 8(5), 385–399, 10.1037/0278-7393.8.5.385.

[bib78] White, S. J., & Staub, A. (2012). The distribution of fixation durations during reading: Effects of stimulus quality. *Journal of Experimental Psychology: Human Perception and Performance,* 38(3), 603–617. 10.1037/a0025338. 21910560

[bib79] Wickham, H. (2016). *ggplot2: Elegant graphics for data analysis*. Berlin, Germany: Springer-Verlag. https://ggplot2.tidyverse.org.

[bib80] Wiley, J. F., & Hedeker, D. (2022). Package “brmsmargins”: Bayesian marginal effects for “brms” models. https://cran.r-project.org/web/packages/brmsmargins/brmsmargins.pdf.

[bib81] Williams, C. C., Perea, M., Pollatsek, A., & Rayner, K. (2006). Previewing the neighborhood: The role of orthographic neighbors as parafoveal previews in reading. *Journal of Experimental Psychology: Human Perception and Performance,* 32(4), 1072–1082, 10.1037/0096-1523.32.4.1072. 16846298

[bib82] Yao, B., Hand, C. J., Miellet, S., & Sereno, S. C. (2025). Parafoveal preview benefits magnified. *Cognition,* 261, 106149, 10.1016/j.cognition.2025.106149. 40279921

[bib83] Yao, B., Scott, G. G., Bruce, G., Monteith-Hodge, E., & Sereno, S. C. (2024). Emotion processing in concrete and abstract words: Evidence from eye fixations during reading. *Cognition and Emotion,* 39(7), 1625–1634, 10.1080/02699931.2024.2367062. 38961837

