# Supplementary material for: Parafoveal preview differentially modulates word frequency and contextual predictability effects during reading
Source: J Vis. 2026 Feb 19;26(2):13. doi: 10.1167/jov.26.2.13 (PMC12924140; doi:10.1167/jov.26.2.13)
Supplement: Supplement 1 [file jovi-26-2-13_s001.pdf]

Item	HF/LF	Target	Length	FPM	HP Cloze	LP Cloze
**1**	HF	face	4	372.51	0.40	0.05
**2**	HF	hair	4	147.74	0.55	0.18
**3**	HF	lake	4	42.89	0.60	0.00
**4**	HF	lips	4	55.21	0.70	0.00
**5**	HF	card	4	52.83	0.85	0.00
**6**	HF	book	4	249.04	0.90	0.35
**7**	HF	neck	4	58.94	0.95	0.15
**8**	HF	theme	5	41.50	0.10	0.00
**9**	HF	fight	5	71.44	0.15	0.00
**10**	HF	trend	5	28.04	0.20	0.00
**11**	HF	coast	5	49.72	0.35	0.00
**12**	HF	horse	5	79.14	0.40	0.05
**13**	HF	phone	5	67.53	0.50	0.20
**14**	HF	short	5	192.09	0.50	0.05
**15**	HF	teeth	5	49.02	0.55	0.00
**16**	HF	happy	5	110.08	0.60	0.00
**17**	HF	dying	5	29.53	0.65	0.25
**18**	HF	tired	5	40.24	0.65	0.45
**19**	HF	stand	5	107.46	0.65	0.05
**20**	HF	print	5	31.67	0.70	0.00
**21**	HF	image	5	80.39	0.75	0.15
**22**	HF	stage	5	169.40	0.75	0.05
**23**	HF	lists	5	30.82	0.80	0.00
**24**	HF	cover	5	112.28	0.80	0.00
**25**	HF	child	5	256.06	0.85	0.00
**26**	HF	dream	5	48.54	0.88	0.00
**27**	HF	clock	5	29.47	0.95	0.00
**28**	HF	grass	5	42.41	0.95	0.00
**29**	HF	hands	5	197.59	0.95	0.00
**30**	HF	light	5	224.99	0.95	0.00
**31**	HF	hall	4	125.80	0.45	0.15
**32**	HF	ball	4	73.60	0.60	0.60
**33**	HF	page	4	105.04	0.60	0.05
**34**	HF	wall	4	124.28	0.70	0.05
**35**	HF	army	4	121.80	0.90	0.20
**36**	HF	roof	4	44.78	0.90	0.25
**37**	HF	park	4	116.67	0.95	0.10
**38**	HF	rural	5	66.24	0.10	0.00
**39**	HF	event	5	111.93	0.10	0.00
**40**	HF	agent	5	46.16	0.20	0.00
**41**	HF	sharp	5	52.78	0.35	0.00
**42**	HF	plant	5	86.87	0.45	0.00
**43**	HF	table	5	207.33	0.50	0.05
**44**	HF	river	5	100.59	0.55	0.00
**45**	HF	video	5	64.79	0.65	0.00
**46**	HF	doors	5	252.46	0.65	0.00
**47**	HF	cells	5	59.13	0.65	0.05
**48**	HF	heart	5	146.09	0.65	0.25
**49**	HF	price	5	190.71	0.65	0.00
**50**	HF	trial	5	68.93	0.70	0.00
**51**	HF	speed	5	80.13	0.70	0.05
**52**	HF	words	5	190.53	0.75	0.00
**53**	HF	rough	5	35.40	0.80	0.00
**54**	HF	story	5	141.00	0.80	0.00
**55**	HF	human	5	210.97	0.80	0.10
**56**	HF	beach	5	40.92	0.90	0.00
**57**	HF	sugar	5	37.40	0.95	0.05
**58**	HF	train	5	81.71	0.95	0.05
**59**	HF	older	5	92.07	0.95	0.00
**60**	HF	nurse	5	35.22	1.00	0.10

Item	HF/LF	Target	Length	FPM	HP Cloze	LP Cloze
**61**	HF	town	4	185.08	0.25	0.00
**62**	HF	boat	4	53.08	0.45	0.00
**63**	HF	road	4	265.81	0.65	0.20
**64**	HF	foot	4	73.92	0.75	0.00
**65**	HF	land	4	216.39	0.80	0.00
**66**	HF	bear	4	58.46	0.90	0.05
**67**	HF	ring	4	60.31	1.00	0.00
**68**	HF	pitch	5	29.74	0.15	0.00
**69**	HF	quick	5	56.04	0.20	0.05
**70**	HF	large	5	361.88	0.35	0.00
**71**	HF	paper	5	166.04	0.40	0.00
**72**	HF	error	5	41.06	0.50	0.00
**73**	HF	sheep	5	29.77	0.55	0.05
**74**	HF	third	5	220.28	0.55	0.20
**75**	HF	cycle	5	34.11	0.60	0.05
**76**	HF	teach	5	28.41	0.65	0.00
**77**	HF	track	5	64.50	0.65	0.00
**78**	HF	royal	5	160.10	0.65	0.00
**79**	HF	staff	5	236.47	0.65	0.00
**80**	HF	knife	5	27.89	0.70	0.25
**81**	HF	forty	5	28.96	0.75	0.00
**82**	HF	white	5	255.27	0.75	0.00
**83**	HF	crowd	5	47.24	0.80	0.60
**84**	HF	adult	5	54.67	0.80	0.00
**85**	HF	rugby	5	35.99	0.85	0.05
**86**	HF	glass	5	104.12	0.90	0.10
**87**	HF	chest	5	39.26	0.95	0.05
**88**	HF	smoke	5	39.47	0.95	0.00
**89**	HF	heavy	5	98.88	0.95	0.15
**90**	HF	radio	5	89.97	1.00	0.05
**91**	HF	spot	4	50.61	0.50	0.15
**92**	HF	baby	4	90.06	0.55	0.00
**93**	HF	hill	4	77.10	0.80	0.00
**94**	HF	bird	4	41.18	0.70	0.15
**95**	HF	home	4	524.78	0.80	0.00
**96**	HF	nose	4	44.06	0.85	0.05
**97**	HF	tree	4	65.52	0.90	0.10
**98**	HF	quiet	5	65.21	0.15	0.05
**99**	HF	start	5	213.12	0.30	0.00
**100**	HF	cheap	5	36.73	0.35	0.05
**101**	HF	store	5	46.56	0.40	0.10
**102**	HF	bread	5	34.91	0.45	0.15
**103**	HF	ships	5	49.07	0.50	0.00
**104**	HF	guard	5	32.52	0.55	0.55
**105**	HF	prize	5	33.43	0.55	0.05
**106**	HF	house	5	512.32	0.60	0.20
**107**	HF	rival	5	28.43	0.65	0.00
**108**	HF	stone	5	86.52	0.65	0.00
**109**	HF	mouth	5	98.52	0.65	0.00
**110**	HF	shirt	5	28.72	0.70	0.35
**111**	HF	uncle	5	36.74	0.75	0.00
**112**	HF	parts	5	127.74	0.80	0.00
**113**	HF	crown	5	56.50	0.78	0.15
**114**	HF	empty	5	59.77	0.85	0.00
**115**	HF	green	5	148.59	0.90	0.00
**116**	HF	proud	5	32.87	0.95	0.10
**117**	HF	plate	5	42.08	0.95	0.35
**118**	HF	girls	5	101.06	0.95	0.00
**119**	HF	music	5	158.07	1.00	0.00
**120**	HF	class	5	189.09	1.00	0.00

Item	HF/LF	Target	Length	FPM	HP Cloze	LP Cloze
**121**	LF	stag	4	3.28	0.20	0.00
**122**	LF	pill	4	6.10	0.40	0.00
**123**	LF	jeep	4	4.87	0.45	0.05
**124**	LF	lime	4	6.87	0.50	0.25
**125**	LF	mint	4	7.20	0.75	0.05
**126**	LF	tram	4	4.46	0.80	0.05
**127**	LF	scab	4	0.58	0.85	0.00
**128**	LF	itchy	5	0.84	0.10	0.00
**129**	LF	froth	5	1.49	0.20	0.00
**130**	LF	shark	5	3.34	0.20	0.00
**131**	LF	salty	5	1.86	0.25	0.00
**132**	LF	elbow	5	11.47	0.30	0.00
**133**	LF	razor	5	4.52	0.40	0.00
**134**	LF	dwarf	5	6.62	0.40	0.00
**135**	LF	disco	5	7.49	0.50	0.00
**136**	LF	thief	5	8.07	0.55	0.05
**137**	LF	towel	5	8.84	0.55	0.00
**138**	LF	fairy	5	8.51	0.65	0.00
**139**	LF	organ	5	13.89	0.70	0.05
**140**	LF	solar	5	14.32	0.70	0.00
**141**	LF	burnt	5	12.24	0.80	0.00
**142**	LF	clown	5	3.86	0.80	0.25
**143**	LF	steep	5	17.22	0.85	0.00
**144**	LF	faint	5	18.43	0.85	0.00
**145**	LF	dizzy	5	4.06	0.90	0.05
**146**	LF	crops	5	17.50	0.90	0.00
**147**	LF	cheek	5	20.14	0.90	0.05
**148**	LF	flask	5	3.16	0.95	0.20
**149**	LF	camel	5	4.22	1.00	0.15
**150**	LF	spoon	5	7.84	1.00	0.00
**151**	LF	tomb	4	6.84	0.25	0.10
**152**	LF	slum	4	3.24	0.30	0.00
**153**	LF	dune	4	1.89	0.50	0.00
**154**	LF	worm	4	6.04	0.65	0.05
**155**	LF	loaf	4	3.98	0.80	0.15
**156**	LF	frog	4	5.08	0.90	0.20
**157**	LF	wolf	4	9.07	1.00	0.25
**158**	LF	sewer	5	2.13	0.15	0.00
**159**	LF	couch	5	5.59	0.15	0.00
**160**	LF	cigar	5	5.21	0.20	0.05
**161**	LF	foxes	5	4.54	0.25	0.00
**162**	LF	alert	5	17.32	0.30	0.00
**163**	LF	foggy	5	1.36	0.35	0.00
**164**	LF	ocean	5	21.39	0.35	0.05
**165**	LF	purse	5	7.13	0.40	0.15
**166**	LF	tweed	5	8.24	0.45	0.05
**167**	LF	peers	5	9.33	0.50	0.00
**168**	LF	lemon	5	12.30	0.55	0.00
**169**	LF	sweat	5	14.33	0.60	0.00
**170**	LF	wiped	5	12.29	0.65	0.00
**171**	LF	jeans	5	12.81	0.65	0.00
**172**	LF	puppy	5	4.79	0.75	0.00
**173**	LF	scalp	5	3.96	0.80	0.10
**174**	LF	pearl	5	7.68	0.85	0.00
**175**	LF	veins	5	8.46	0.90	0.05
**176**	LF	altar	5	9.86	0.90	0.00
**177**	LF	fence	5	16.72	0.90	0.00
**178**	LF	pizza	5	3.68	0.95	0.00
**179**	LF	witch	5	6.59	1.00	0.00
**180**	LF	chalk	5	9.80	1.00	0.00

Item	HF/LF	Target	Length	FPM	HP Cloze	LP Cloze
**181**	LF	maze	4	5.08	0.30	0.00
**182**	LF	reef	4	5.48	0.50	0.00
**183**	LF	doll	4	7.28	0.65	0.00
**184**	LF	mall	4	3.11	0.75	0.00
**185**	LF	wand	4	1.53	0.80	0.05
**186**	LF	cork	4	9.52	0.85	0.05
**187**	LF	dice	4	3.54	0.95	0.05
**188**	LF	igloo	5	0.20	0.10	0.00
**189**	LF	toxic	5	13.10	0.15	0.00
**190**	LF	brass	5	15.97	0.20	0.00
**191**	LF	punch	5	15.54	0.25	0.10
**192**	LF	salsa	5	0.17	0.30	0.10
**193**	LF	drums	5	7.32	0.40	0.00
**194**	LF	faded	5	15.34	0.45	0.00
**195**	LF	sober	5	6.68	0.50	0.00
**196**	LF	shiny	5	7.67	0.55	0.10
**197**	LF	desks	5	3.81	0.55	0.00
**198**	LF	onion	5	6.57	0.60	0.00
**199**	LF	twins	5	12.53	0.60	0.00
**200**	LF	vague	5	15.89	0.60	0.30
**201**	LF	stain	5	5.26	0.65	0.00
**202**	LF	feast	5	9.54	0.65	0.10
**203**	LF	tribe	5	8.06	0.70	0.10
**204**	LF	ankle	5	10.41	0.75	0.00
**205**	LF	dusty	5	8.28	0.85	0.00
**206**	LF	ivory	5	9.77	0.85	0.25
**207**	LF	fizzy	5	1.06	0.90	0.00
**208**	LF	stamp	5	14.23	0.90	0.00
**209**	LF	fibre	5	16.64	0.95	0.20
**210**	LF	beast	5	9.87	1.00	0.15
**211**	LF	dome	4	7.10	0.10	0.00
**212**	LF	herd	4	7.43	0.35	0.15
**213**	LF	lice	4	1.83	0.55	0.00
**214**	LF	slug	4	2.24	0.60	0.00
**215**	LF	pier	4	5.78	0.75	0.00
**216**	LF	yolk	4	1.11	0.80	0.00
**217**	LF	moth	4	2.99	0.80	0.25
**218**	LF	muddy	5	6.70	0.20	0.00
**219**	LF	barge	5	3.50	0.25	0.00
**220**	LF	chewy	5	0.32	0.25	0.10
**221**	LF	shaky	5	5.08	0.30	0.00
**222**	LF	acorn	5	2.59	0.40	0.00
**223**	LF	album	5	23.01	0.40	0.00
**224**	LF	spade	5	3.03	0.45	0.00
**225**	LF	stack	5	6.98	0.45	0.00
**226**	LF	ditch	5	6.78	0.50	0.05
**227**	LF	stale	5	4.33	0.55	0.00
**228**	LF	cakes	5	10.61	0.60	0.05
**229**	LF	skull	5	12.34	0.60	0.05
**230**	LF	lions	5	9.96	0.70	0.23
**231**	LF	beard	5	9.74	0.70	0.00
**232**	LF	claws	5	3.16	0.80	0.05
**233**	LF	piano	5	20.21	0.80	0.05
**234**	LF	snail	5	2.99	0.85	0.20
**235**	LF	steak	5	4.32	0.85	0.10
**236**	LF	salad	5	10.86	0.85	0.00
**237**	LF	scary	5	1.94	0.90	0.00
**238**	LF	zebra	5	2.21	0.90	0.00
**239**	LF	cloud	5	23.07	0.95	0.45
**240**	LF	grave	5	23.11	1.00	0.40
