# Supplementary material for: Parafoveal preview differentially modulates word frequency and contextual predictability effects during reading
Source: J Vis. 2026 Feb 19;26(2):13. doi: 10.1167/jov.26.2.13 (PMC12924140; doi:10.1167/jov.26.2.13)
Supplement: Supplement 2 [file jovi-26-2-13_s002.pdf]

Supplementary Materials B: Experimental Materials and Counterbalancing

Experimental materials comprised a total of 240 two-line passages of text. Half of the passages contained high frequency (HF) targets and half contained length-matched low frequency (LF) targets. Each HF or LF target appeared in the second sentence of each passage and was preceded by a context sentence whose content was biasing or neutral with respect to the upcoming target. Thus, target words were deemed to be either high or low in predictability (HP, LP), respectively. Finally, the parafoveal preview of the target was manipulated, with either the target word appearing as itself in readers' pre-target fixations (Valid preview) or appearing as a visually similar pronounceable nonword that was replaced by the target once readers' eyes crossed over the last letter of the pre-target word (Invalid preview). This 2 (Frequency: HF, LF) × 2 (Predictability: HP, LP) × 2 (Preview: Valid, Invalid) design gave rise to 8 experimental conditions.

The complete set of all experimental passages are presented in **Table B1**. Items 1-120 contain HF targets and items 121-240 contain LF targets. Target words are underlined. Both HP and LP context sentences are listed before each target sentence, although any given participant was presented with only one of the contexts. Invalid previews, although only utilized in half of the conditions, are shown in parentheses preceding target words.

Four non-overlapping lists of materials were constructed so that each participant read all HF and all LF passages, but with each passage in only one of its 4 possible conditions (HP-Valid, HP-Invalid, LP-Valid, LP-Invalid). The counterbalancing of items and conditions across these 4 presentation lists of materials is presented in **Table B2**. Across all lists, each participant was presented with 30 passages in each of the 8 conditions in a randomized order.

Each set of 30 passages were roughly equal to each other in terms of their lexical specifications. The mean target word length, frequency, and predictability values (with *SD*s) for each set of 30 items are presented in **Table B3**.

Table B1

Experimental Passages

Item	Sentence	Pred	HF Targets
**1**	Context	HP	Simon was very careful when he shaved before his job interview.
		LP	Simon used his arms to brace his fall on the pavement.
	Target		An unsightly cut on his (toin)<u>face</u> would look bad to his employers.
**2**	Context	HP	Jessica's cat was moulting over all the furniture in her flat.
		LP	Jessica started cleaning her flat by dusting the surfaces.
	Target		The sofa was covered in (tine)<u>hair</u> and needed to be vacuumed.
**3**	Context	HP	The fisherman began to row his little boat on his fishing trip.
		LP	He was determined to return with a prize worthy of a top champion.
	Target		When he got the middle of the (bula)<u>lake</u> he cast his line.
**4**	Context	HP	The referee's watch said time was up and he raised his whistle.
		LP	The amateur referee ran to the traffic cone located near the pitch.
	Target		Placing it to his (tayn)<u>lips</u> he gave a loud blast when the game ended.
**5**	Context	HP	I often make mistakes when giving people their birthday gifts.
		LP	People always say that I'm absent-minded but well-intentioned.
	Target		Forgetting to sign the (mesk)<u>card</u> is normally my biggest mistake.

6	Context	HP	Trips on the train can be very boring without something to do.
		LP	Some people keep entertained on long trips by watching DVDs.
	Target		Remember to take a good (fand)book and dull journeys will seem shorter.
7	Context	HP	The consensus was that the serial strangler had struck again.
		LP	The examination was carried out as a matter of normal procedure.
	Target		The marks around the (crat)neck confirmed the detective's conclusion.
8	Context	HP	Everyone knew that “EastEnders” was just beginning.
		LP	We were so busy baking cakes in the kitchen we had forgotten the time.
	Target		We recognised the familiar (blorn)theme tune and sat down to watch.
9	Context	HP	Dave and Gordon watched the boxers in the match exchange blows.
		LP	Dave and Gordon were going to watch the match on the HDTV in the pub.
	Target		Afterwards, they agreed that the (dryth)fight was very exciting.
10	Context	HP	The analysts found a tendency for increased spending in the recession.
		LP	The Gulf air would bring warmer temperatures to the UK.
	Target		It was forecast that this (leask)trend would continue for a while.
11	Context	HP	One advantage of Britain being an island is the fabulous beaches.
		LP	There are many popular tourist destinations in France.
	Target		It is very common for people to visit the (wrick)coast for a holiday.
12	Context	HP	More bets are placed on the Grand National than any other race.
		LP	Sporting events are often wagered upon to win money.
	Target		People choose the (frour)horse they think will win and place their bets.
13	Context	HP	I was dozing on the sofa as I waited for a call from the plumber.
		LP	I was dozing on the sofa and nearly fell asleep.
	Target		All of a sudden, the (gleas)phone rang and completely startled me.
14	Context	HP	There was a height restriction to get on the rollercoaster.
		LP	At the funfair, the group of friends raced towards the Ghost Train.
	Target		Some of the kids were too (cleck)short to go on the ride.
15	Context	HP	Sarah had saved money to have veneers fitted at the dentist.
		LP	Sarah paid for a hospital where she would have private clinicians.
	Target		When they were finished, her (frilt)teeth looked fabulous.
16	Context	HP	The couple finally got pregnant after trying for months.
		LP	The teenagers tried for weeks to get into the boarded up building.
	Target		They were extremely (degip)happy when they eventually succeeded.
17	Context	HP	The vet examined the critically ill dog in her surgery.
		LP	Meg approached the bird that was caught in the net.
	Target		She tried to prevent it from (agorp)dying but it was too late.
18	Context	HP	The farmer worked hard all day in his fields.
		LP	Ian’s first day at work had been just as eventful as he’d hoped.
	Target		He was extremely (brean)tired when he came home.
19	Context	HP	The toddler held onto the furniture to keep himself upright.
		LP	The old man’s health had begun to deteriorate at an increasing pace.
	Target		On his own, he was unable to (choul)stand without falling down.
20	Context	HP	Marvin had to go to the shops to buy a new ink cartridge.
		LP	Marvin couldn’t meet up with his friends to brag about his trip.
	Target		At present, he was unable to (gowel)print of his colour photos.
21	Context	HP	Dan was traumatised by seeing the mutilated body as a child.
		LP	Dan’s phobia had been caused by a snake biting him as a child.
	Target		He could never get rid of the (serpo)image from his mind's eye.
22	Context	HP	The famous soprano received a standing ovation from the audience.
		LP	The memorial ceremony for Michael Jackson was beautiful.
	Target		People threw flowers at the (chups)stage to show their admiration.

**23**	Context	HP	Sheena had to shop for many things in many different stores.
		LP	The teacher prepared for the classes she would have next week.
	Target		She made up several (frade)lists so that she remembered everything.
**24**	Context	HP	The painters were told not to damage any of the furniture.
		LP	The artists were commissioned to design a mural for the lobby.
	Target		Before they began, they had to (acrom)cover everything with sheets.
**25**	Context	HP	Parents must ensure appropriate supervision in their absence.
		LP	The cooker is one appliance where safety is a priority.
	Target		It is important never to leave a (stoft)child unattended in the kitchen.
**26**	Context	HP	The night after her day at the zoo, Natalie fell into a deep sleep.
		LP	Natalie’s parents told her they would be living in Africa next year.
	Target		She had a very unusual (hower)dream about being chased by a chimp.
**27**	Context	HP	Susan was bored in the lecture and time passed slowly.
		LP	Susan’s guidance counsellor had many curious objects on his shelves.
	Target		She kept looking at the (shest)clock and counted down the minutes.
**28**	Context	HP	The park keepers took good care of the lawns.
		LP	The hotel owners were expecting external evaluators to arrive any day.
	Target		They made sure that the (pance)grass was cut every day.
**29**	Context	HP	It was a cold day and Barbara had forgotten her gloves.
		LP	Last night, Barbara went for a walk to think about moving house.
	Target		She decided to keep her (trule)hands in her pockets for warmth.
**30**	Context	HP	Seth could easily carry six plastic chairs at a time.
		LP	The minister invested in durable banquet tables for the church hall.
	Target		They were incredibly (fryth)light and could be stacked together.
**31**	Context	HP	The class of 300 students had arrived to sit their final exam.
		LP	They had been revising hard for the British Citizenship exam.
	Target		Everybody in the (loth)hall was determined to do well in the test.
**32**	Context	HP	Robbie and his dad were getting ready to play catch.
		LP	Edward and Gillian had been searching for over an hour.
	Target		They finally found the (teth)ball in the back corner of the front closet.
**33**	Context	HP	The librarian was disgraced at the damage to the returned book.
		LP	The student unwillingly handed in his jotter to be graded.
	Target		There wasn't a single (gopa)page without a tear or smudge.
**34**	Context	HP	Crashing the car into the living room left Jim with a large bill.
		LP	The work was scheduled to take place over the winter months.
	Target		Rebuilding the (muth)wall would be the most expensive part of the job.
**35**	Context	HP	Many young boys dream of becoming soldiers when they grow up.
		LP	Kids are often asked what they would like to be when they grow up.
	Target		A career with the (narp)army seems an exciting adventure to children.
**36**	Context	HP	The man we had hired to replace our slates had a fatal accident.
		LP	The man’s family were consoled that he had not suffered in agony.
	Target		The fall from the (mant)roof had killed him instantly on impact.
**37**	Context	HP	We decided to take the children to play on the swings on Sunday.
		LP	The children waited eagerly in the car for their parents.
	Target		A trip to the (yeat)park is a lovely treat when the sun is shining.
**38**	Context	HP	Many people are opting to leave cities for a quieter life.
		LP	Not everybody lives near their place of work.
	Target		They move to (creef)rural areas and commute to work instead.
**39**	Context	HP	George found a marquee to host his son's wedding reception.
		LP	George was told the mountain top cabin provided spectacular outlooks.
	Target		It was ideal for the (nouch)event so he hired it immediately.

**40**	Context	HP	The Ministry of Defence discovered a spy in their operation.
		LP	The tour guide arranged and led art history trips to Eastern Europe.
	Target		He was a Russian (nysol)agent who was relaying details to Moscow.
**41**	Context	HP	The knife was blunt and Nigel was struggling to cut the turkey.
		LP	Nigel began to shape the marble sculpture with a small chisel.
	Target		He asked his wife for a (clurg)sharp one and continued to carve.
**42**	Context	HP	Terry went to the new gardening centre.
		LP	Terry had never been to Stirling’s out-of-town shopping centre.
	Target		He bought a rare (ghock)plant for his garden.
**43**	Context	HP	The young couple were wanting to furnish their new dining room.
		LP	The older couple visited the store on the high street.
	Target		They selected a (futir)table that was exactly what they wanted.
**44**	Context	HP	People sometimes tried to drown themselves by jumping off the bridge.
		LP	The dreadful car accident happened at 6am when the roads were quiet.
	Target		A boy was pulled from the (cusic)river by a passer-by walking their dog.
**45**	Context	HP	The DVD is now the most common form of movie entertainment.
		LP	Many things are being replaced by more modern innovations.
	Target		It seems that the (antom)video will soon be a thing of the past.
**46**	Context	HP	Mark's car was damaged by the side-on crash at the junction.
		LP	Mark was annoyed when he realised how much he would have to spend.
	Target		He would need new (bream)doors before his car was roadworthy.
**47**	Context	HP	The thugs were arrested and brought to the police station.
		LP	The owners of the castle knew what to do with unruly peasants.
	Target		They put them in the (ratir)cells overnight as punishment.
**48**	Context	HP	Doctors warn against excess cholesterol and promote exercise.
		LP	School age children are taught about the importance of their health.
	Target		Looking after the (brock)heart is an important task for all age groups.
**49**	Context	HP	Inflation commonly rises by a small percentage every year.
		LP	The MP asked for additional anti-terrorism measures to be imposed.
	Target		The result is an increase in the (quain)price of goods that we purchase.
**50**	Context	HP	The lawyers were behind schedule in selecting the jurors.
		LP	Kyle and Simone were waiting for a call from the company.
	Target		They were hoping to begin the (favid)trial as quickly as possible.
**51**	Context	HP	The yacht crew were pleased with the favourable strong wind.
		LP	The new route would help them arrive earlier than planned.
	Target		They used it to gain (nysol)speed and were able to win the race.
**52**	Context	HP	David increased his vocabulary by reading lots of books.
		LP	David was glad he had studied moral philosophy at university.
	Target		His knowledge of difficult (miate)words was far better than others.
**53**	Context	HP	The joiner hadn't smoothed the edges of the cabinets yet.
		LP	The carpenter had just finished staining the wooden doors.
	Target		They were still quite (sumpt)rough and not ready to be varnished.
**54**	Context	HP	Johnny liked his father to read to him before bedtime.
		LP	Johnny enjoyed his first day at primary school.
	Target		There was one particular (chung)story he liked about a tiger.
**55**	Context	HP	Only when they had no choice, the cannibals would eat monkey flesh.
		LP	The aliens had strange customs and eating habits.
	Target		They preferred the taste of (frove)human flesh over animals.
**56**	Context	HP	I love the feeling of sand under my feet and the sound of waves.
		LP	I often take my children out on the weekends.
	Target		Going to the (trest)beach to collect shells is an enjoyable activity.

**57**	Context	HP	Ted was diabetic and had to monitor what he ate.
		LP	Ted had to monitor his diet carefully for his heart condition.
	Target		If he ate too much (rapon)sugar he could become unwell.
**58**	Context	HP	Stuart did not want to travel to London by bus or plane.
		LP	Stuart wanted to see his favourite band in London.
	Target		He bought tickets for the (deece)train to Waterloo on the Internet.
**59**	Context	HP	Matthew’s younger sister was born several years after him.
		LP	Her brother, Matthew, was working towards his black belt in karate.
	Target		Because he is (uthos)older than her, he is protective.
**60**	Context	HP	The patient had been cared for in the hospital for weeks.
		LP	His long stay was not as tiresome as he had thought it would be.
	Target		He had a favourite (searn)nurse who looked after him.
**61**	Context	HP	Keith was known to be a terrible but well-meaning local gossip.
		LP	Aunt Lillian calls all her relatives on a regular basis.
	Target		If something happens, the entire (bine)town will know within hours.
**62**	Context	HP	Tour Pleasure Cruises were ideal for people who enjoyed diving.
		LP	The children liked to visit their rich uncle’s coastal villa.
	Target		They would jump off the (krid)boat into the cool blue water.
**63**	Context	HP	The emergency services were attending to a terrible car crash.
		LP	The vehicle had been set on fire in the multi-storey car park.
	Target		The police closed the (sart)road until the wreckage was removed.
**64**	Context	HP	Tom had accidentally walked on a piece of glass at the beach.
		LP	Tom had an injury but he had to remain in uniform all day long.
	Target		He finally examined the cut on his (dech)foot and it looked infected.
**65**	Context	HP	Farms are often very valuable to potential property developers.
		LP	Their new investment would mean they could retire early.
	Target		They will use the (trab)land to build new homes and make lots of money.
**66**	Context	HP	Our holiday in the Canadian wilderness came to a terrifying end.
		LP	Our militia took cover in the hills but it did not go as planned.
	Target		Our camp was attacked by a (dace)bear that had smelled our food.
**67**	Context	HP	Men who are proposing often spend many hours in jeweller's shops.
		LP	The father gave his son some timely advice.
	Target		Choosing the right (semp)ring may make the difference in the outcome.
**68**	Context	HP	Much care went into watering the field before the football match.
		LP	The rival teams were due to play at the refurbished sports complex.
	Target		On the day of the game, the (yolet)pitch looked better than ever.
**69**	Context	HP	Jean couldn’t stay long at her father’s because she was running late.
		LP	Jean had to wait a few minutes before Greg came to the door.
	Target		She was only paying him a (pinel)quick visit to see if he was ok.
**70**	Context	HP	Their spacious lounge could easily accommodate thirty people.
		LP	Their lounge required major refurbishment.
	Target		It was very (femps)large with high ceilings and a fireplace.
**71**	Context	HP	The secretary sliced the tip of her finger on the letter.
		LP	Harriet’s hands smarted and stung as she was doing the recycling.
	Target		She hated getting these (joyac)paper cuts and swore loudly.
**72**	Context	HP	Daphne's computer wasn't letting her open the application.
		LP	Daphne followed the instructions down to the very last detail.
	Target		She kept getting (amone)error messages and called the support line.
**73**	Context	HP	The shepherd had spotted a wolf prowling around his fields.
		LP	The man heard that a neighbour’s goats had been savaged by a predator.
	Target		He kept a close eye on his (clury)sheep to protect his flock.

**74**	Context	HP	Last year, Frank narrowly missed the bronze and came in fourth.
		LP	Frank worked furiously to submit his art portfolio on time.
	Target		This year, he hoped to finish at least (kloat)third in the competition.
**75**	Context	HP	In cities, there are often special bus and taxi lanes.
		LP	To make our roads less busy, it is advised to walk short journeys.
	Target		Sometimes there are also (squim)cycle lanes to ease traffic.
**76**	Context	HP	Ms. Hart had the flu and needed her classes to be covered.
		LP	Linda’s whole face had become swollen from the infection.
	Target		She would be unable to (frint)teach for at least a week.
**77**	Context	HP	There had been a terrible crash at the weekend's Grand Prix.
		LP	An unstable canister had fallen off one of the maintenance lorries.
	Target		Oil had leaked onto the (foust)track and caused a massive pile-up.
**78**	Context	HP	The Queen has never voted in a General Election.
		LP	Several politicians will join the anti-war demonstration.
	Target		Members of the (miped)royal family are not allowed to.
**79**	Context	HP	Mood around the office was glum and the boss needed to take action.
		LP	The manager of the private nursery had extra funds to spend.
	Target		He decided to organise an event for the (chelt)staff to boost morale.
**80**	Context	HP	Craig knew the law about carrying illegal weapons in public.
		LP	A while ago, Craig’s parents had grounded him for a whole month.
	Target		He still carried a (timba)knife despite the risk of being caught.
**81**	Context	HP	Melanie and Danielle shared the eighty jelly beans evenly.
		LP	Melanie and Danielle shared the big bag of sweets between them.
	Target		Each girl received (brelp)forty sweets and ate them greedily.
**82**	Context	HP	During apartheid in South Africa, most races could not vote.
		LP	In the past, politics was controlled by the elite in society.
	Target		Only people who were (stods)white could take part in the elections.
**83**	Context	HP	Tiger Woods was angry when he was distracted playing a shot.
		LP	Fred felt he would have an advantage playing in the home ground.
	Target		Apparently, someone in the (sneck)crowd cheered as he hit the ball.
**84**	Context	HP	Little Peter was still afraid to cross the road by himself.
		LP	Tom was rebuilding the motor on his car but was having some problems.
	Target		He needed the aid of a competent (elath)adult to help him with the task.
**85**	Context	HP	Henry had been injured in a scrum at school.
		LP	Henry had injured himself during a routine warm-up at the gym.
	Target		He was unable to play (siple)rugby for several weeks.
**86**	Context	HP	Rob liked the pub’s drink specials but disliked their hygiene.
		LP	Rob ordered a baguette and a pint from the pub for lunch.
	Target		He noticed that his (phrew)glass was cracked and told the waitress.
**87**	Context	HP	The pirates located the spot where the treasure was buried.
		LP	Stan and his gang had taken what wasn’t rightfully theirs.
	Target		They opened up the (sload)chest and marvelled at the booty inside.
**88**	Context	HP	Jennifer tried a cigarette for the first time and loved it.
		LP	Jennifer had discovered a new way of talking to strangers.
	Target		She started to regularly (varts)smoke when she went out.
**89**	Context	HP	Marcus almost hurt himself badly at the gym lifting weights.
		LP	The delivery of birdbaths for Marcus’ garden shop arrived.
	Target		He had picked ones that were too (drisp)heavy for him to lift.
**90**	Context	HP	Jared had forgotten to bring CDs on his long car journey.
		LP	Jared knew he should revise for his test which was just next week.
	Target		Instead, he turned the (anter)radio on and tuned in a rock station.

**91**	Context	HP	The teenager had broken out in terrible acne.
		LP	His back had become a bit swollen and ached a little.
	Target		There was one particular (wyal)spot that needed to be squeezed.
**92**	Context	HP	New parents often find it impossible to get a full night's sleep.
		LP	We hadn’t realised how thin the walls were at the holiday resort.
	Target		The noise of a (felp)baby crying in the next room makes sleeping hard.
**93**	Context	HP	The cyclist was overcome by the steepness of the incline.
		LP	The disabled man was having trouble coping with everyday life.
	Target		He couldn’t get up the (keft)hill as his as legs were too painful.
**94**	Context	HP	Our house cat loves to pounce on unsuspecting sparrows in the garden.
		LP	The farmer’s cat was getting old but was still a great hunter.
	Target		Going out to catch a (foat)bird was a treat the cat looked forward to.
**95**	Context	HP	Gran had become very senile and couldn't look after herself.
		LP	Our new budgie was in danger of being eaten by our cat.
	Target		We decided to put her in a (leam)home where she would be less vulnerable.
**96**	Context	HP	The drunken man was taken to hospital after being head-butted.
		LP	Darren’s parents took him to A&E and he was finally admitted.
	Target		The doctor repaired his broken (vern)nose and prescribed painkillers.
**97**	Context	HP	Our day of kite flying was cut short when it became stuck.
		LP	The wet blanket from the boat was on the line drying in the breeze.
	Target		It got caught in a (hain)tree and we had to climb up and retrieve it.
**98**	Context	HP	The burglar wore soft shoes to avoid being heard.
		LP	Paul often helped himself to sweets once his mum left the kitchen.
	Target		He was always very (praik)quiet and had never been caught.
**99**	Context	HP	Harry hated missing the beginning of films at the cinema.
		LP	Harry was always running late for every occasion.
	Target		Once again, he had missed the (whurl)start of the movie and was annoyed.
**100**	Context	HP	At the end of season sale, prices were much reduced.
		LP	She was surprised at the quality of the items in the vintage store.
	Target		The clothes were (slony)cheap but still of very high quality.
**101**	Context	HP	Shoppers were excited about the clothes shop being built in town.
		LP	The odd design of the exterior of the business drew much attention.
	Target		When it was opened, the new (cleam)store attracted many customers.
**102**	Context	HP	Every morning, Jeff would walk past the baker's shop.
		LP	Jeff liked to visit the summer market in the town centre.
	Target		He enjoyed the smell of (faunt)bread and frequently bought a loaf.
**103**	Context	HP	During the War, German submarines targeted supply convoys.
		LP	During the war, the enemy forces caused distress and destruction.
	Target		They would attack the (aboge)ships that carried weapons and food.
**104**	Context	HP	We approached the prison camp where our friend was being held captive.
		LP	It was getting dark and we made our final approach with great care.
	Target		In the distance we saw a (peast)guard pacing back and forth with a rifle.
**105**	Context	HP	Tony wanted to win in this year's maths competition.
		LP	Tony hoped that he would soon be able to call himself Mr. Universe.
	Target		He wanted the (garem)prize money more than anything else.
**106**	Context	HP	The young couple were eager to get onto the property market.
		LP	The average teenager reaches many milestones after leaving school.
	Target		Deciding to buy a (drern)house is one of life's big commitments.
**107**	Context	HP	The gang leader had been gunned down as he left his house.
		LP	The police spokesman read out a statement about the kidnapping.
	Target		It was done by members of a (wrach)rival gang in a revenge attack.

**108**	Context	HP	I could feel something in my shoe which dug into my heel.
		LP	The child ran home with something he had taken from the garden.
	Target		It was a small (chran)stone which had come from the gravel path.
**109**	Context	HP	I didn't realise how hot my curry was as I ate a big spoonful.
		LP	After my public talk, my nerves were completely frazzled.
	Target		The peculiar sensation in my (smoll)mouth only went away with a few beers.
**110**	Context	HP	The assistant at the bank spilled ink all down his front.
		LP	Tom gorged himself on the freshly picked blueberries.
	Target		This left a stain on his (cload)shirt and he was angry at himself.
**111**	Context	HP	Jack's aunt was supposed to pick him up after school.
		LP	After riding the rollercoaster, Jack looked for his brother.
	Target		Instead, it was his (vands)uncle who was waiting for him.
**112**	Context	HP	The plumber couldn't mend the boiler until next week.
		LP	Paul decided to build a cabin to house his hunting equipment.
	Target		He had to order the (quafe)parts he needed from a specialist shop.
**113**	Context	HP	The Archbishop of Canterbury presided over the King's coronation.
		LP	At the end of the cartoon, the wizard turned the frog back into a man.
	Target		When he placed the (unrew)crown on his head, the ceremony was complete.
**114**	Context	HP	Liz and her friends polished off all the food in her flat.
		LP	Liz was friends with the workmen who were remodelling her kitchen.
	Target		The refrigerator was (rejip)empty after they left.
**115**	Context	HP	The army designed new camouflage to be used in forests.
		LP	The novelty birthday cake was shaped like a mountain range.
	Target		It was mostly dark (porve)green but had patches of black and brown.
**116**	Context	HP	Maria's only son was graduating today from Oxford.
		LP	Maria was meeting with the Headmaster when he summoned in her son.
	Target		As she watched him, she felt so (queck)proud of his achievements.
**117**	Context	HP	Mary's young son gave her a kick as she washed the dishes.
		LP	Mary suddenly noticed a fox staring at her from her back window.
	Target		She was so surprised, she dropped a (ghuba)plate and it smashed.
**118**	Context	HP	At school, Miss Jones told only the boys to leave early.
		LP	The Mother Superior noticed that all the communion wine was gone.
	Target		She wanted to talk to the (quate)girls about the incident.
**119**	Context	HP	Keith liked to listen to Mozart, the Beatles, and techno.
		LP	Keith was undecided which genre he should choose for his essay.
	Target		He liked all kinds of (siver)music with no particular preference.
**120**	Context	HP	Adam’s behaviour at school was getting out of control.
		LP	Tricia told her line manager about her difficulty working with Adam.
	Target		He kept disrupting the (stoin)class and got into fights during break times.

Item	Sentence	Pred	LF Targets
**121**	Context	HP	I thought that a nice set of antlers would look good in my study.
		LP	I had finally thought of the perfect gift for my father-in-law.
	Target		I went out to hunt for a (choy)stag that would make a good trophy buck.
**122**	Context	HP	The doctor had prescribed me medicine for my angina.
		LP	The doctor said I was run down and should take some time off.
	Target		He told me to take a (yoth)pill with water after my evening meal.
**123**	Context	HP	The safari hunter startled his prey when he fired a shot at it.
		LP	The vet went to get medicine for the sick animal at the park.
	Target		He hurried to his (pory)jeep and sped off after the lion.
**124**	Context	HP	The hot sun shone on the pool bar as I ordered a gin and tonic.
		LP	The bowl of punch has lots of fruit in it and I ordered a glass.
	Target		It came with a wedge of (bown)lime and loads of ice.
**125**	Context	HP	I have a friend who is always stuffing his face with chewing gum.
		LP	I have a friend who is always stuffing his face with sweets.
	Target		There is a strong smell of (neek)mint whenever John opens his mouth.
**126**	Context	HP	Planners are building a new public transport system in Edinburgh.
		LP	With congestion, councils must redesign towns to aid commuters.
	Target		Constructing a (bewe)tram system is a useful way of easing traffic.
**127**	Context	HP	Ian's wound was almost completely healed so he decided to pick it.
		LP	Ian couldn’t wait to see how it looked underneath.
	Target		He carefully pulled the (rext)scab and felt satisfied when it came off.
**128**	Context	HP	Andrea constantly suffered from severe eczema.
		LP	Andrea had an appointment to see a doctor at the clinic.
	Target		She was always (shalp)itchy and constantly scratched her arms.
**129**	Context	HP	Maude added two brown sugars to her cappuccino.
		LP	Maude ordered ice cubes for her milkshake at McDonald’s.
	Target		She put her spoon through the (drelk)froth and stirred them in.
**130**	Context	HP	Swimmers in the sea spotted the distinctive fin and called out.
		LP	The lifeguards raised the alarm at the tourist resort.
	Target		Everyone hurried from the (clouf)shark as soon as the warning went out.
**131**	Context	HP	Brad and Phoebe bought a large box of popcorn at the movies.
		LP	Brad and Phoebe both ordered the fish curry at the restaurant.
	Target		However, it was too (metig)salty so they didn’t eat much of it.
**132**	Context	HP	Nadia had been practising her tennis stroke for six hours.
		LP	Nadia had been practising most of the afternoon.
	Target		She now had a pain in her (ottun)elbow and went to get an ice pack.
**133**	Context	HP	David's toilet bag was searched for sharp objects at the airport.
		LP	David was travelling through Europe and decided to go to a festival.
	Target		Security confiscated his (minan)razor and he'd have to buy a new one.
**134**	Context	HP	Jamie loved basketball but he was very short for his age.
		LP	Ian enrolled in the Dutch school when his parents moved to Amsterdam.
	Target		In gym class, he felt like a (frout)dwarf next to his classmates.
**135**	Context	HP	My parents met in the Seventies and loved to go out dancing.
		LP	The couple spent a lot of their free time together.
	Target		Every weekend they would go to a (bowen)disco and dance the night away.
**136**	Context	HP	The shopkeeper suspiciously eyed the girl in the hooded top.
		LP	George decided that the next time she came in, he would do something.
	Target		He knew she was a (klead)thief and hoped to catch her red-handed.

**137**	Context	HP	After his morning jog, Gregor was happy to take a long, hot shower.
		LP	In the morning, Gregor forgot where he had last put his contacts.
	Target		When he stepped out, he reached for his (frest)towel but it wasn’t there.
**138**	Context	HP	If Cinderella was to go to the ball, she would need a miracle.
		LP	Nina joked that she required a makeover to look good for the party.
	Target		She needed the help of her (toing)fairy godmother to make her over.
**139**	Context	HP	Maria carried a donor card in case she was in an accident.
		LP	Maria correctly filled in her medical details on the hospital forms.
	Target		Doctors could use any (amper)organ in the event of her death.
**140**	Context	HP	The sun's heat can be used as a renewable source of energy.
		LP	Rural communities are often encouraged to upgrade their houses.
	Target		People can use (raken)solar panels on their roofs for power.
**141**	Context	HP	Zoe had a habit of forgetting to check food in the oven.
		LP	Zoe found life difficult when she moved out of her parents’ house.
	Target		Most of the time she (frask)burnt her meals and had to start over.
**142**	Context	HP	Pierre had entertained kids at the circus for fifty years.
		LP	Pierre loved to make people laugh and had made a career of it.
	Target		He had enjoyed being a (skane)clown but it was time to retire.
**143**	Context	HP	The amateur cyclists found it difficult to ascend the hill.
		LP	The boy scouts made their way through the forest in the downpour.
	Target		The path was (whang)steep so they took several breaks.
**144**	Context	HP	When Geoffrey got a nosebleed, Dawn nearly keeled over.
		LP	When her boyfriend got into a fight, Dawn became quite upset.
	Target		We thought she was going to (broud)faint after seeing all the blood.
**145**	Context	HP	At the ceilidh, Steven vigorously spun Emma round and round.
		LP	Steven dared Emma to run across the bridge stretching above the river.
	Target		This made her very (brimp)dizzy but she still had a good time.
**146**	Context	HP	Farmer Joe dusted his fields to combat the insect infestation.
		LP	Steve was not impressed by the antics of the local hooligans.
	Target		They had started to attack his (snage)crops and he had to act quickly.
**147**	Context	HP	Maria’s boyfriend had been drinking and he slapped her in the face.
		LP	Maria saw the football coming towards her and tried to dodge it.
	Target		She could feel her left (stoud)cheek redden and started to cry.
**148**	Context	HP	The window cleaner always carried a supply of hot tea.
		LP	Luke was always drinking green tea at work.
	Target		He kept it in a (thund)flask that he filled up every morning.
**149**	Context	HP	Ponies and horses are not suited to travelling across deserts.
		LP	Some animals have always been used for transportation.
	Target		The best animal for this is the (seach)camel as it rarely needs water.
**150**	Context	HP	Emily had never seen such an enormous bowl of ice cream.
		LP	Emily was famished and didn’t care who was watching.
	Target		She excitedly grabbed a (equim)spoon and began to stuff herself.
**151**	Context	HP	Egyptian pharaohs were often given extravagant burial chambers.
		LP	The history lecturer talked about leaders from ancient civilisations.
	Target		Building an ornate (fank)tomb was customary to celebrate each ruler.
**152**	Context	HP	The aid worker visited the poorest and dirtiest part of the city.
		LP	The private sector built more housing for the expanding population.
	Target		Many people lived in the (edor)slum and their conditions were terrible.
**153**	Context	HP	I stood at the bottom of the sandy slope and prepared to run.
		LP	When I returned home, I felt like going for an outdoor sprint.
	Target		I ran to the top of the (larn)dune and felt a burning in my calves.

**154**	Context	HP	Experts say using live bait will improve your angling success.
		LP	On our camping trip, we avidly followed advice from specialist books.
	Target		Placing a fresh (rece)worm on the end of the line should attract a fish.
**155**	Context	HP	Panic struck when I ran out of bread making the kids' sandwiches.
		LP	I was making Lea's lunch when I remembered her friend was coming too.
	Target		I hurried out to get a (bant)loaf so I could finish making the lunches.
**156**	Context	HP	The witch cast a spell to transform the prince into another form.
		LP	In the fantasy novel, the android Kyra attacked commander Devlin.
	Target		Turning him into a (tump)frog left her free to take over the kingdom.
**157**	Context	HP	Everyone knows what happens at the end of Little Red Riding Hood.
		LP	The thriller's turning point is when some animals are found dead.
	Target		The woodsman kills the (vutt)wolf with an axe and the story ends well.
**158**	Context	HP	Waste had overflowed onto the road and the smell was awful.
		LP	The minor tremor caused much damage to our neighbourhood.
	Target		Luckily, workers repaired the broken (ranom)sewer within two hours.
**159**	Context	HP	The young couple were shopping for new living room furniture.
		LP	The young couple had extra money from their wedding to spend.
	Target		They purchased a new (meast)couch that had very soft upholstery.
**160**	Context	HP	Sebastien's holiday to Cuba had been the trip of a lifetime.
		LP	The old gentleman had become accustomed to fine wine and dining.
	Target		He often enjoyed a (zepia)cigar after dinner.
**161**	Context	HP	Hounds used for hunting are trained in special kennels.
		LP	Some farmers train their dogs to do special tasks.
	Target		They are taught to chase (bence)foxes out of their burrows.
**162**	Context	HP	Fiona always had two cups of strong coffee to wake her up.
		LP	Fiona used her fruit juicer relentlessly every morning.
	Target		This made her feel more (skack)alert and ready to tackle the day.
**163**	Context	HP	Will heard that a large mist was forecasted to move inland that night.
		LP	Will was driving fast to meet his friends for that night's game.
	Target		It suddenly became (boppa)foggy as Will drove and he had to slow down.
**164**	Context	HP	The couple took the cruise ship across the Pacific to Hawaii.
		LP	The day was hot and the couple made their way upstairs.
	Target		The breeze from the (varew)ocean kept them cool on deck.
**165**	Context	HP	After paying for her groceries, the cashier gave Lisa her change.
		LP	Lisa checked that she had her key before leaving her flat.
	Target		She put it in her (yamor)purse and carried the bags to her car.
**166**	Context	HP	The old professor dressed as a stereotypical academic.
		LP	When she met her blind date, she chuckled at his fashion sense.
	Target		His jacket was (drack)tweed and had patches on the elbows.
**167**	Context	HP	Ryan's friends influenced him to drink at the school disco.
		LP	Ryan parents wanted to know why he quit his new job.
	Target		It was because of pressure from his (grize)peers that he did it.
**168**	Context	HP	At their local pub, the office workers all ordered gin and tonics.
		LP	The restaurant staff were extremely busy with two birthday parties.
	Target		The manager started to cut up a (haver)lemon for the drinks.
**169**	Context	HP	Lorna had gone on a five mile run in the midday sun.
		LP	Lorna had managed to build up her flat-pack wardrobe by herself.
	Target		You could see the (canch)sweat running down her face by the end.
**170**	Context	HP	The toddler ran up to his parents with his face covered in snot.
		LP	The toddler was distraught about his broken toy.
	Target		His mum leaned over and (majit)wiped his nose with a soft tissue.

**171**	Context	HP	The new store carried the latest range of denim clothing.
		LP	Kate had been chatting online to Jim for many weeks on a dating site.
	Target		Kate treated herself to expensive (gruce)jeans for her big date.
**172**	Context	HP	Emma prayed her parents would get her a cute pet this Christmas.
		LP	Emma asked for just one present when she wrote her letter to Santa.
	Target		Her heart leapt when she saw a beautiful (jogyr)puppy sitting patiently.
**173**	Context	HP	Sidney had tried a new shampoo for his terrible dandruff.
		LP	Sidney tried the oil the pharmacist had recommended to him.
	Target		He massaged it into his (noady)scalp before rinsing it out well.
**174**	Context	HP	Gavin was diving for oysters and got lucky when he opened one.
		LP	Gavin was happy to return from his snorkelling holiday.
	Target		He had found a large (queck)pearl and gave it to his wife.
**175**	Context	HP	Heroin addicts often tie a belt tightly around their arms.
		LP	Doctors often apply pressure to certain regions of the body.
	Target		This allows them to locate some (naron)veins that they inject into.
**176**	Context	HP	The priest smiled as the bride and groom exchanged their vows.
		LP	Tim couldn’t believe that Jean was on time for once.
	Target		They stood at the (ethem)altar and looked deeply into each other's eyes.
**177**	Context	HP	The neighbour’s Alsatian kept coming into Valerie’s garden.
		LP	Valerie recently took in a stray dog from the shelter.
	Target		She got her son to build a (krean)fence to keep it away from her roses.
**178**	Context	HP	Peter liked extra cheese and mushrooms as toppings.
		LP	Flying first class to America, Peter was asked if he wanted anything.
	Target		He ordered a large (grice)pizza with a side of wedges.
**179**	Context	HP	Old Mrs. Greeble was warty, haggard, and had a fearsome black cat.
		LP	Miss Dearborn lived at number 31 in Alder Crescent.
	Target		The older kids said that she was a (redal)witch to scare the younger ones.
**180**	Context	HP	The teacher scrawled the sentences onto the blackboard.
		LP	The teacher turned back to the lesson after scolding the kids.
	Target		The noise of the (stutt)chalk sent shivers up everyone's spine.
**181**	Context	HP	Theseus used string to guide his way through the minotaur's lair.
		LP	Brian loved to explore but he always took precautions.
	Target		Getting back out the (weam)maze would be easier if he left a trail.
**182**	Context	HP	Our Pacific dive gave us the opportunity to see natural coral.
		LP	The old shipwreck was a favourite setting for several reasons.
	Target		Swimming around the (surk)reef was a wonderful way to see exotic fish.
**183**	Context	HP	My sister used to play with girly toys and had a clear favourite.
		LP	My grandmother fondly recounted stories about when she was young.
	Target		As a child, there was one (neft)doll she refused to be without.
**184**	Context	HP	On holiday in America, we spent Saturday shopping for clothes.
		LP	My friends and I decided to spend Monday together.
	Target		We drove to the (soth)mall on the outskirts of town and had a good day.
**185**	Context	HP	The good witch used magic to make children's wishes come true.
		LP	The children loved when the teacher enacted the stories.
	Target		She would take out a (mesk)wand and wave it about.
**186**	Context	HP	Traditional wine producers do not like screw caps on wine bottles.
		LP	Traditionalists prefer natural products for wine production.
	Target		They think the best substance to use is (nand)cork instead of plastic.
**187**	Context	HP	I needed to roll a double six with my last throw to win the game.
		LP	I am rather competitive and always want to beat my opponent!
	Target		I picked up the (torm)dice and said a silent prayer as I threw them.

**188**	Context	HP	The Eskimo family hunted for weeks to prepare for the Arctic winter.
		LP	The family hunted for weeks to prepare for the approaching winter.
	Target		They stocked their (spizz)igloo with enough food to last months.
**189**	Context	HP	The nuclear plant had contaminated the area with noxious waste.
		LP	The surveyors spent several weeks in an area outside of town.
	Target		The land was very (bisco)toxic and could not be used for decades.
**190**	Context	HP	The band included trumpets, trombones, French horns and tubas.
		LP	The band included several members of the same family.
	Target		It was the most famous (hiver)brass ensemble in Canada.
**191**	Context	HP	The boys got into a fist fight in the playground.
		LP	The two squabbling boys were finally left unsupervised on the bus.
	Target		They began to furiously (yexel)punch each other in the face.
**192**	Context	HP	After rhumba and tango classes, the pair wanted to try something new.
		LP	The friends were deciding which evening class they should take.
	Target		They thought that the (antis)salsa class would be the most fun.
**193**	Context	HP	In music class, Ricky discovered that he had natural rhythm.
		LP	Tim was new to the class and his teacher needed to assess his level.
	Target		His teacher sat him at the (hover)drums and told him to play away.
**194**	Context	HP	After many washes, Karl's shirt had lost most of its colour.
		LP	The dog’s blanket in the back of Karl’s truck had seen better days.
	Target		It was so badly (tefal)faded that he needed to buy a replacement.
**195**	Context	HP	Dr. Adams was still drunk when he was due to start work.
		LP	Dr. Adams made his way into work, despite feeling under the weather.
	Target		He would need to (anlom)sober up quickly or he would be sacked.
**196**	Context	HP	Robert was polishing his shoes before his big job interview.
		LP	Robert was dismayed to see that the ornaments had gathered dust.
	Target		He wanted them to be (whemp)shiny enough to see his face in them.
**197**	Context	HP	Before the new school year, all the furniture was replaced.
		LP	The council awarded a small grant to the high school’s library.
	Target		Pupils would have new (boafe)desks that were free from graffiti.
**198**	Context	HP	Alison's eyes were watering as she chopped the vegetables.
		LP	Alison normally steamed her food but today she was in a hurry.
	Target		She added the (vazir)onion and peppers into the oil in the pan.
**199**	Context	HP	The pregnant girl's family had a history of multiple births.
		LP	The girl had expected her appointment to be straightforward.
	Target		The nurse told her she had (bowe)twins when she went for her scan.
**200**	Context	HP	The witness did not get a good look at the mugger.
		LP	The police wanted to use her account to narrow down the suspects.
	Target		Her description was (niper)vague and not very helpful.
**201**	Context	HP	Jill's friends were drinking red wine all night in her flat.
		LP	Jill shuddered as the rain battered against her doors and windows.
	Target		In the morning, she noticed an enormous (whone)stain on the carpet.
**202**	Context	HP	The triumphant King arranged a sumptuous and lavish banquet.
		LP	Sir Blakewell smiled as he recalled the events from that evening.
	Target		It was a delightful (druch)feast which was heartily devoured by all.
**203**	Context	HP	The anthropologist studied the ways of different African peoples.
		LP	Last year, Ray travelled around Africa with his camcorder.
	Target		Each month he filmed a different (durko)tribe to record their customs.
**204**	Context	HP	Karen had jumped and landed awkwardly while ice skating.
		LP	Someone spiked the volleyball directly at Karen in gym class.
	Target		She badly hurt her (velts)ankle and would need an x-ray.

**205**	Context	HP	I couldn't stop sneezing as I cleaned out the storage room.
		LP	I had offered to tidy up the sitting room in my student flat.
	Target		Everything was (boulp)dusty and got up my nose as I worked.
**206**	Context	HP	Poachers still illegally hunt elephants for their tusks.
		LP	It is illegal to hunt endangered species but some disobey this.
	Target		It is possible to buy (crung)ivory items on the black market.
**207**	Context	HP	The bottle of coke had been opened a few days ago.
		LP	Liam’s friend handed him a drink at the party.
	Target		Liam drank some, but it was not (dromp)fizzy and tasted bad.
**208**	Context	HP	The letter Lucas had posted was returned to him.
		LP	Lucas had hastily written a cheque to his window cleaner.
	Target		He had forgotten to put a (ching)stamp on it before posting it.
**209**	Context	HP	Maintaining a healthy digestive system requires roughage.
		LP	Some breakfast cereals are actually not very healthy.
	Target		Foods that are high in (tober)fibre are recommended by experts.
**210**	Context	HP	In the Disney film, Belle falls in love with the castle’s monster.
		LP	The fairytale ends happily as would be expected.
	Target		The village beauty and the (tarch)beast live happily ever after.
**211**	Context	HP	An arena was built in London to mark the new millennium.
		LP	The citizens were not pleased to see what their taxes had paid for.
	Target		Everyone agreed that the (brin)dome was a terrible waste of money.
**212**	Context	HP	The elephant family prepared to travel to their destination.
		LP	The tourists watched the animals departing through their binoculars.
	Target		The members of the (lunk)herd set off, trunk to tail, on their journey.
**213**	Context	HP	Al's head was itching terribly and one look revealed the problem.
		LP	Al begged his mum to let him stay home from school.
	Target		He had caught (fren)lice from one of the other children at school.
**214**	Context	HP	Pouring salt around the creature that eats my lettuces is pleasing.
		LP	The bait is placed in the container and set on the ground at night.
	Target		This traps the (whep)slug and I take perverse pleasure in its demise.
**215**	Context	HP	We tearfully said our goodbyes to Tommy as he boarded the ferry.
		LP	We said our goodbyes to Tommy as he was leaving the neighbourhood.
	Target		We stood and waved at the end of the (gron)pier as the ship left port.
**216**	Context	HP	Betty only needed an egg white to make her meringue nest.
		LP	Betty disliked wasting food when she was baking.
	Target		Later, she used the (geth)yolk to make a separate dish.
**217**	Context	HP	It is important to protect clothes from being eaten by insects.
		LP	You have to be careful not to leave clothes outside overnight.
	Target		A determined and hungry (zelt)moth could ruin an entire wardrobe.
**218**	Context	HP	The school football team persevered despite the wet and dirty pitch.
		LP	The school football team had travelled a long way to play the match.
	Target		The children returned home (selig)muddy but happy to have won.
**219**	Context	HP	The gypsies travelled along the canal in the middle of the night.
		LP	Gypsies were illegally trying to get into the neighbouring country.
	Target		They hid in the cargo of a slow moving (demys)barge afraid of discovery.
**220**	Context	HP	Flo couldn't eat the sticky toffee because of her dentures.
		LP	When her young grandson gave her a sweet, Flo eagerly ate it.
	Target		It was far too (stimp)chewy and got stuck to her false teeth.
**221**	Context	HP	Sean suffered from the symptoms of Parkinson's Disease.
		LP	Sean had been lifting weights all morning and he could see the effects.
	Target		His arms were (chelp)shaky and his family was worried.

**222**	Context	HP	The grey squirrel was foraging at the foot of the oak tree.
		LP	Sammy often hid things in the back garden for safekeeping.
	Target		He recovered the (narem)acorn that he had buried last winter.
**223**	Context	HP	The record company wanted Tara to record some new songs.
		LP	Tara’s company were putting pressure on her to work harder.
	Target		They wanted her next (otlar)album to come out before Christmas.
**224**	Context	HP	Rory was going to dig all day in the potato fields.
		LP	Rory’s wife made him sandwiches for his long day ahead.
	Target		He picked up his (equir)spade and headed off to work.
**225**	Context	HP	Luke's first job was working at the supermarket.
		LP	Luke’s first job wasn’t exciting but at least he would earn money.
	Target		His responsibility was to (clomb)stack the shelves.
**226**	Context	HP	The driver hadn’t seen the trench that had been dug to drain water.
		LP	The driver sped past the countryside house carelessly.
	Target		The car crashed into the (fedul)ditch and had to be written off.
**227**	Context	HP	Leon was unhappy with the tough bread he got with his soup.
		LP	Leon was unhappy with the coffee he was served in a UK cafe.
	Target		He complained that it was (cluts)stale and the waitress apologised.
**228**	Context	HP	Tina's mother was baking in the kitchen.
		LP	Tina was preparing a picnic for their family outing.
	Target		She made lots of (rebra)cakes for the whole family to enjoy.
**229**	Context	HP	The cause of death was a hammer blow to the head.
		LP	The results from the investigation were recorded on the certificate.
	Target		The damage to the victim's (cleth)skull was quite sickening.
**230**	Context	HP	Everyone was excited about going to see the big cats at the zoo.
		LP	The teachers had organised a trip to Blair Drummond’s adventure park.
	Target		The children wanted to see (doric)lions and tigers most of all.
**231**	Context	HP	Albert thought he looked good with his new facial hair.
		LP	Albert thought his new look made him look just like David Beckham.
	Target		His friends disagreed and thought his (trunt)beard looked awful.
**232**	Context	HP	Eve's cat had begun to scratch her new furniture.
		LP	Eve was in charge of the injured leopard at the zoo.
	Target		She would need to get its (stoun)claws cut to prevent further damage.
**233**	Context	HP	The music teacher hired removal men when he moved house.
		LP	Simon hired removal men when he moved house.
	Target		He couldn't move his (gream)piano alone because it was too heavy.
**234**	Context	HP	When I visited Paris, I tried a well-known French delicacy.
		LP	I went to a restaurant and ordered something I had never tried.
	Target		I scooped out the inside of a (crink)snail and swallowed it whole.
**235**	Context	HP	The Big Ranch restaurant's speciality was high quality beef.
		LP	He was known to have a healthy appetite.
	Target		Bill ordered a huge (choul)steak and a pitcher of beer.
**236**	Context	HP	Tania first prepared the tomatoes, cucumber and lettuce.
		LP	Tania was surprised at how quickly her guests devoured the appetisers.
	Target		She finished making the (nitch)salad with oil and vinegar dressing.
**237**	Context	HP	The child couldn't sleep after watching the monster movie.
		LP	The young child had been to the fun fair for the first time.
	Target		It had been really (wrimp)scary and she was afraid to be alone.
**238**	Context	HP	The children were confused by a horse with stripes on its side.
		LP	The children were adamant that they should ride on the horse.
	Target		I explained that it was actually a (miden)zebra and they seemed content.

**239**	Context	HP	It was a lovely summer's day until the sun went away.
		LP	We were gazing up at Chicago's skyscrapers when the sun vanished.
	Target		It disappeared behind a (stest)<u>cloud</u> and it became colder.
**240**	Context	HP	Frank's wife died in giving birth to their son.
		LP	Frank still missed not having his mother around.
	Target		He would visit her (pream)<u>grave</u> on Sundays.

Table B2

Counterbalancing of Experimental Conditions by Item Number across Presentation Lists 1 to 4

	HF				LF			
	HP		LP		HP		LP	
	Valid	Invalid	Valid	Invalid	Valid	Invalid	Valid	Invalid
**List 1**	1-30	31-60	61-90	91-120	121-150	151-180	181-210	211-240
**List 2**	31-60	1-30	91-120	61-90	151-180	121-150	211-240	181-210
**List 3**	61-90	91-120	1-30	31-60	181-210	211-240	121-150	151-180
**List 4**	91-120	61-90	31-60	1-30	211-240	181-210	151-180	121-150

Note: HF = high frequency; LF = low frequency; HP = high predictability; LP = low predictability; Valid = valid preview; Invalid = invalid preview.

Table B3

Mean Target Word Frequency and Predictability Specifications (with *SD*s) by Item Groupings

		1-30		31-60		61-90		91-120	
**HF**	**Length**	4.77	(0.43)	4.77	(0.43)	4.77	(0.43)	4.77	(0.43)
	**Frequency**	102.29	(86.35)	103.71	(58.64)	105.44	(90.97)	103.73	(122.86)
	**HP Cloze**	0.65	(0.25)	0.66	(0.25)	0.67	(0.24)	0.68	(0.22)
	**LP Cloze**	0.07	(0.12)	0.07	(0.12)	0.06	(0.12)	0.09	(0.13)

		121-150		151-180		181-210		211-240	
**LF**	**Length**	4.77	(0.43)	4.77	(0.43)	4.77	(0.43)	4.77	(0.43)
	**Frequency**	7.78	(5.50)	8.06	(4.89)	8.32	(4.95)	7.51	(6.71)
	**HP Cloze**	0.61	(0.27)	0.60	(0.28)	0.61	(0.25)	0.61	(0.25)
	**LP Cloze**	0.04	(0.07)	0.04	(0.07)	0.05	(0.08)	0.07	(0.12)

Note: Units are as follows: Length in number of characters; Frequency in occurrences per million; Cloze as a probability that the target word was correctly guessed within the given context. HF = high frequency; LF = low frequency; HP = high predictability; LP = low predictability.
