# Supplementary material for: Parafoveal preview differentially modulates word frequency and contextual predictability effects during reading
Source: J Vis. 2026 Feb 19;26(2):13. doi: 10.1167/jov.26.2.13 (PMC12924140; doi:10.1167/jov.26.2.13)
Supplement: Supplement 3 [file jovi-26-2-13_s003.pdf]

Supplementary Materials C: Model Diagnostics

Prior Predictive Check

We extracted prior samples from our BMMs (using *sample_prior = "only"* in brms) to examine their distributional properties.

For each dataset, we calculated the median and scaled Median Absolute Deviation (MAD; comparable to *SD* in a normal distribution) of simulated fixation durations and the predicted skipping rate, enabling us to assess the plausibility of prior predictions. With consistent priors across all analyses, the prior simulations exhibited highly overlapping distributions for different fixation measures. The simulated data revealed theoretically plausible, positively skewed quasi-Gaussian distributions, with median fixation durations around 225 ms, ranging from approximately 100 to 400 ms, and median skipping probability (PrSkip) around 0.25, ranging from approximately 0.05 to 0.50. The scaled MAD of simulated samples ranged from 30 to 125 ms, indicating appropriate rightward shifts and increased skew in later measures (**Figure C1**). The scaled MAD of the predicted skipping probability ranged from approximately 0.05 to 0.45, with a plausible positive skew reflecting more extreme variations across subjects and items (**Figure C1**).

Figure C1

Prior Predictive Checks for Fixation Measures

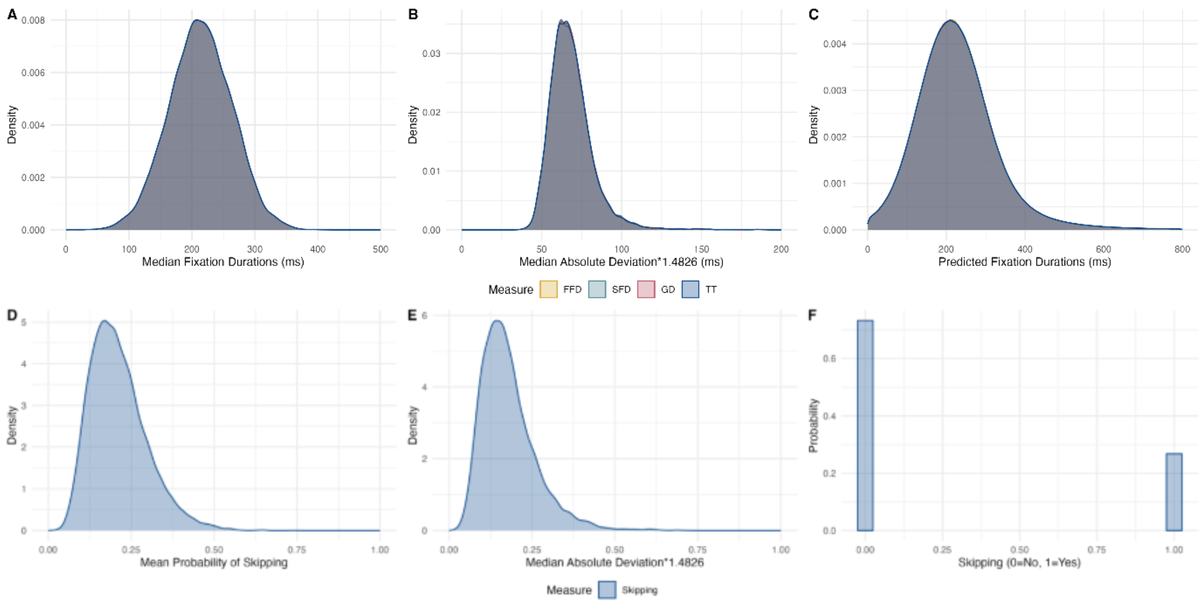

Note: In the first row, the left panel (A) illustrates the distributions of median fixation durations. The middle panel (B) shows the distributions of the Median Absolute Deviation (MAD) of fixation durations, scaled by 1.4826 to be equivalent to the standard deviation for normal distributions. The right panel (C) illustrates the prior distributions of fixation durations. In the second row, the left panel (D) illustrates the distribution of median skipping probability. The middle panel (E) shows the distribution of the 1.48*MAD of skipping probability. The right panel (F) illustrates the prior predictive distribution of skipping outcomes (0 = not skipped; 1 = skipped).

The prior parameter estimates for fixed effects reflect our prior specifications (**Table C1**). All simulations used identical seeds for reproducibility, resulting in highly consistent parameters across analyses, with minor variations reflecting the specific data structure of each fixation measure after pre-processing.

These checks demonstrate that our prior specifications generated realistic fixation duration patterns and appropriate parameter ranges, providing strong validation for our modelling approach.

Table C1

Prior-Only Parameter Estimates for Fixed Effects

Factor<br>Measure	*Est.b(mu)*	*Est.Err*	$CrI_{2.5}$	$CrI_{97.5}$	*Est.b(beta)*	*Est.Err*	$CrI_{2.5}$	$CrI_{97.5}$
**Intercept**								
**FFD**	225.11	49.54	127.95	321.29	4.09	0.22	3.66	4.52
**SFD**	225.11	49.54	127.89	321.28	4.09	0.22	3.66	4.51
**GD**	225.11	49.54	127.95	321.29	4.09	0.22	3.66	4.52
**TT**	225.11	49.54	127.93	321.31	4.09	0.22	3.66	4.52
**PrSkip**	-1.39.	0.50	-2.38	-0.40				
**Frequency**								
**FFD**	-0.05	15.33	-30.35	29.92	0	0.24	-0.48	0.46
**SFD**	-0.05	15.33	-30.35	29.92	0	0.24	-0.48	0.46
**GD**	-0.05	15.33	-30.35	29.92	0	0.24	-0.48	0.46
**TT**	-0.05	15.33	-30.35	29.92	0	0.24	-0.48	0.46
**PrSkip**	0.00	0.30	-0.58	0.58				
**Predictability**								
**FFD**	-0.18	14.9	-29.15	28.68	0	0.24	-0.47	0.47
**SFD**	-0.18	14.9	-29.15	28.68	0	0.24	-0.47	0.47
**GD**	-0.18	14.9	-29.15	28.68	0	0.24	-0.47	0.47
**TT**	-0.18	14.9	-29.15	28.68	0	0.24	-0.47	0.47
**PrSkip**	0.00	0.30	-0.58	0.57				
**Preview**								
**FFD**	-0.21	15.03	-29.69	29.37	0	0.24	-0.47	0.46
**SFD**	-0.21	15.03	-29.69	29.37	0	0.24	-0.47	0.46
**GD**	-0.21	15.03	-29.69	29.37	0	0.24	-0.47	0.46
**TT**	-0.21	15.03	-29.69	29.37	0	0.24	-0.47	0.46
**PrSkip**	0.00	0.30	-0.59	0.59				
**Frequency × Predictability**								
**FFD**	0.12	15.00	-28.91	29.05	0	0.24	-0.47	0.48
**SFD**	0.12	15.00	-28.91	29.05	0	0.24	-0.47	0.48
**GD**	0.12	15.00	-28.91	29.05	0	0.24	-0.47	0.48
**TT**	0.12	15.00	-28.91	29.05	0	0.24	-0.47	0.48
**PrSkip**	0.00	0.29	-0.57	0.57				
**Frequency × Preview**								
**FFD**	-0.13	15.24	-29.98	29.94	0	0.24	-0.46	0.47
**SFD**	-0.13	15.24	-29.98	29.94	0	0.24	-0.46	0.47
**GD**	-0.13	15.24	-29.98	29.94	0	0.24	-0.46	0.47
**TT**	-0.13	15.24	-29.98	29.94	0	0.24	-0.46	0.47
**PrSkip**	0.00	0.30	-0.59	0.59				

**Predictability × Preview**								
**FFD**	0.06	15.02	-29.37	29.15	0	0.24	-0.47	0.47
**SFD**	0.06	15.02	-29.37	29.15	0	0.24	-0.47	0.47
**GD**	0.06	15.02	-29.37	29.15	0	0.24	-0.47	0.47
**TT**	0.06	15.02	-29.37	29.15	0	0.24	-0.47	0.47
**PrSkip**	0.00	0.30	-0.58	0.59				
**Frequency × Predictability × Preview**								
**FFD**	0.06	14.98	-28.88	29.12	0	0.24	-0.48	0.48
**SFD**	0.06	14.98	-28.88	29.12	0	0.24	-0.48	0.48
**GD**	0.06	14.98	-28.88	29.12	0	0.24	-0.48	0.48
**TT**	0.06	14.98	-28.88	29.12	0	0.24	-0.48	0.48
**PrSkip**	0.00	0.30	-0.60	0.59				

Note: Summary statistics (estimate, standard error, and 95% credible intervals) from prior predictive simulations, showing the distribution of model parameters before observing any data. FFD = first fixation duration; SFD = single fixation duration; GD = gaze duration; TT = total fixation time; PrSkip = skipping probability.

Posterior Predictive Check

To validate our models' ability to capture empirical patterns in the data, we extracted posterior samples (using *posterior_predict()*) and compared their distributions against observed fixation durations. Posterior predictions show excellent correspondence with the observed data distributions across all measures (**Figure C2**), with highly overlapping density and bar plots demonstrating that our models successfully captured both the central tendencies and the distributional characteristics of the empirical data. This close alignment between model predictions and observed patterns provides strong evidence for the adequacy of our BMMs in capturing empirical characteristics of the observed data.

Figure C2

Posterior and Observed Data Distributions across Fixation Measures

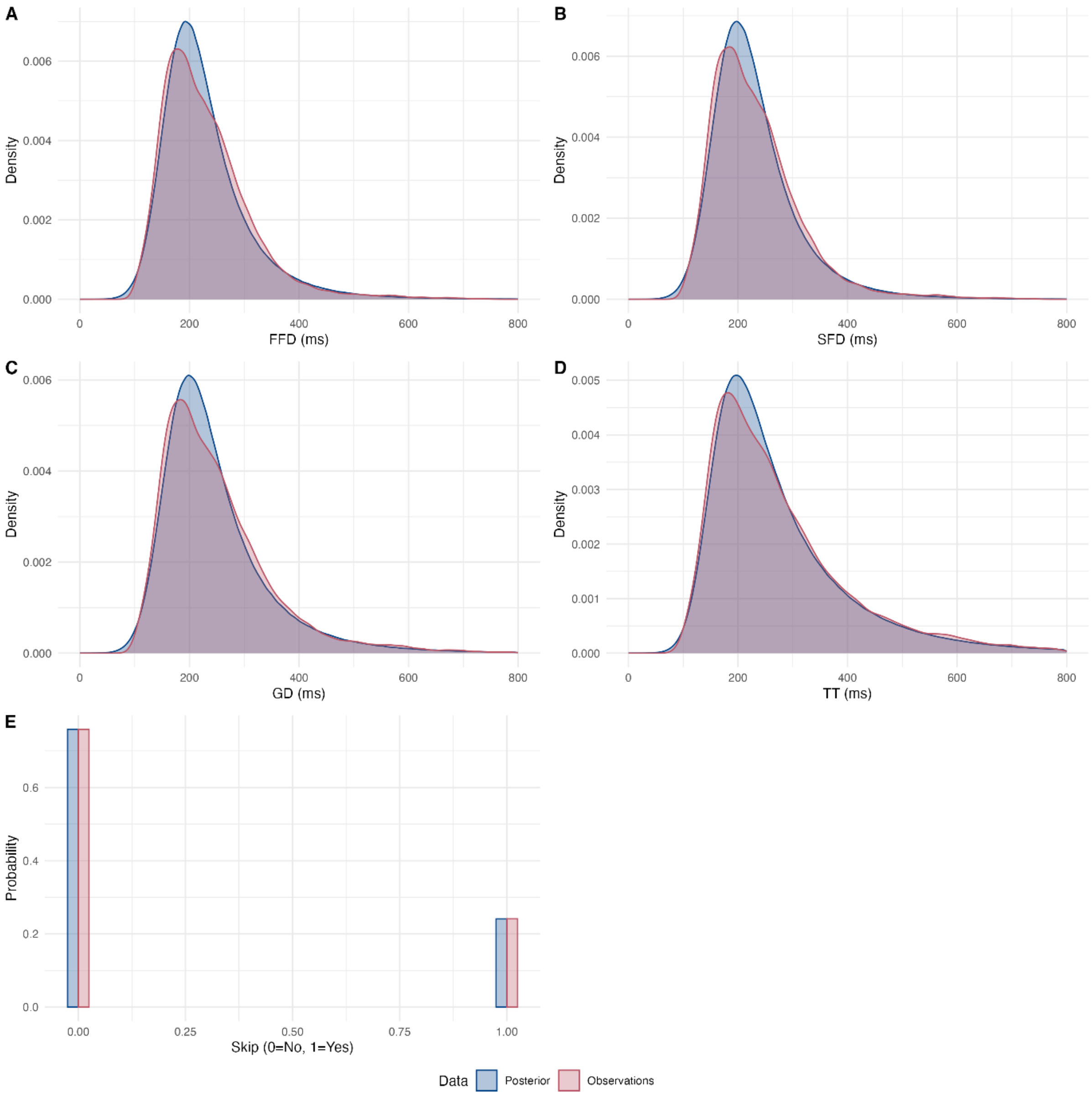

Note: Panels (A), (B), (C), and (D) represent posterior and observed data density functions for FFD, SFD, GD, and TT fixation measures, respectively. Panel (E) displays posterior and observed skipping probability in bar charts. FFD = first fixation duration; SFD = single fixation duration; GD = gaze duration; TT = total fixation time.
