# Supplementary material for: Parafoveal preview differentially modulates word frequency and contextual predictability effects during reading
Source: J Vis. 2026 Feb 19;26(2):13. doi: 10.1167/jov.26.2.13 (PMC12924140; doi:10.1167/jov.26.2.13)
Supplement: Supplement 4 [file jovi-26-2-13_s004.pdf]

## Supplementary Materials D: Controlling for Launch Distance

The launch distance to a target word is defined as the number of characters from the pre-target fixation to the space preceding the target word. It has been used as a proxy measure of the degree of target parafoveal preview benefit, with longer distances providing a reduced parafoveal preview and shorter ones providing an enhanced preview (see, e.g., Hand et al., 2010).

However, two important caveats apply. First, launch distance serves as a meaningful proxy for preview availability only under valid preview conditions (i.e., during normal reading). In the present experiment, parafoveal processing was explicitly manipulated using invalid previews, which disrupt the typical facilitative relationship between target proximity and preview benefit. Consequently, the interpretation of launch distance as an index of preview benefit is not straightforward under these antagonistic conditions. Second, launch distance is confounded by target skipping. When a target is skipped, no launch distance is recorded for that item. Under valid preview conditions, easier targets (e.g., higher frequency, higher predictable words) are more likely to be skipped when the eyes are closer to the target, which artificially inflates the mean launch distance for those conditions. Together, these issues make launch distance a potentially misleading covariate in analyses that include both valid and invalid preview conditions, particularly when skipping behavior differs systematically across conditions.

Nonetheless, we included launch distance (≤ 20 characters) as a fixed factor interacting with Frequency, Predictability, and Preview in four Bayesian mixed models for fixation duration measures. This control addressed whether the observed Frequency × Preview and Predictability × Preview interactions on GD persisted when accounting for lower-level oculomotor variation.

**Table D1**

**BMM Results by Fixed Effects and Fixation Measures**

| Factor / Measure | *b(mu)* | $CrI_{2.5}$ | $CrI_{97.5}$ | $BF_{10}$ | *b(beta)* | $CrI_{2.5}$ | $CrI_{97.5}$ | $BF_{10}$ |
|---|---|---|---|---|---|---|---|---|
| **Intercept** | | | | | | | | |
| **FFD** | 227.83 | 221.86 | 233.93 | | 4.07 | 3.99 | 4.14 | |
| **SFD** | 230.63 | 224.28 | 237.09 | | 4.03 | 3.95 | 4.10 | |
| **GD** | 245.02 | 237.20 | 252.82 | | 4.23 | 4.15 | 4.31 | |
| **TT** | 277.40 | 267.62 | 286.87 | | 4.66 | 4.59 | 4.73 | |
| **Frequency** | | | | | | | | |
| **FFD** | **-4.51** | **-7.49** | **-1.51** | **7.33** | **-0.11** | **-0.16** | **-0.06** | **201.63** |
| **SFD** | **-5.60** | **-8.94** | **-2.25** | **27.34** | **-0.13** | **-0.19** | **-0.07** | **>1000** |
| **GD** | **-7.57** | **-11.25** | **-3.98** | **>1000** | **-0.12** | **-0.17** | **-0.07** | **>1000** |
| **TT** | **-7.85** | **-12.64** | **-3.20** | **34.00** | **-0.08** | **-0.12** | **-0.04** | **68.99** |
| **Predictability** | | | | | | | | |
| **FFD** | **-5.65** | **-8.16** | **-3.12** | **>1000** | *-0.06* | *-0.11* | *-0.01* | *1.86* |
| **SFD** | **-6.30** | **-9.18** | **-3.46** | **>1000** | *-0.06* | *-0.12* | *-0.01* | *1.17* |
| **GD** | **-7.34** | **-10.24** | **-4.47** | **>1000** | **-0.10** | **-0.15** | **-0.05** | **183.29** |
| **TT** | **-15.77** | **-20.01** | **-11.66** | **>1000** | **-0.11** | **-0.15** | **-0.07** | **>1000** |

| | | | | | | | | |
|---|---|---|---|---|---|---|---|---|
| **Preview** | | | | | | | | |
| **FFD** | **-40.91** | **-45.41** | **-36.55** | **>1000** | **-0.42** | **-0.48** | **-0.36** | **>1000** |
| **SFD** | **-45.74** | **-50.87** | **-40.71** | **>1000** | **-0.40** | **-0.47** | **-0.33** | **>1000** |
| **GD** | **-55.49** | **-61.70** | **-49.51** | **>1000** | **-0.47** | **-0.53** | **-0.41** | **>1000** |
| **TT** | **-69.16** | **-76.98** | **-61.33** | **>1000** | **-0.41** | **-0.46** | **-0.35** | **>1000** |
| **Frequency × Predictability** | | | | | | | | |
| **FFD** | -0.50 | -5.62 | 4.60 | 0.16 | 0.02 | -0.08 | 0.12 | 0.24 |
| **SFD** | -0.68 | -6.52 | 5.28 | 0.21 | -0.01 | -0.13 | 0.11 | 0.25 |
| **GD** | 1.80 | -3.86 | 7.56 | 0.23 | 0.06 | -0.04 | 0.16 | 0.44 |
| **TT** | -0.15 | -8.16 | 7.99 | 0.28 | 0 | -0.09 | 0.08 | 0.18 |
| **Frequency × Preview** | | | | | | | | |
| **FFD** | *6.05* | *0.59* | *11.50* | *1.82* | 0.08 | -0.02 | 0.17 | 0.76 |
| **SFD** | *6.44* | *0.61* | *12.38* | *1.90* | 0.05 | -0.06 | 0.16 | 0.32 |
| **GD** | **8.89** | **2.89** | **14.90** | **15.94** | 0.08 | -0.01 | 0.17 | 0.87 |
| **TT** | *8.30* | *0.77* | *15.92* | *2.82* | 0.03 | -0.04 | 0.12 | 0.24 |
| **Predictability × Preview** | | | | | | | | |
| **FFD** | *-6.06* | *-11.04* | *-1.02* | *2.27* | 0 | -0.10 | 0.10 | 0.21 |
| **SFD** | *-6.04* | *-11.57* | *-0.42* | *1.74* | 0.04 | -0.07 | 0.16 | 0.31 |
| **GD** | **-8.76** | **-14.22** | **-3.25** | **27.21** | -0.02 | -0.11 | 0.08 | 0.21 |
| **TT** | -4.92 | -12.14 | 2.23 | 0.62 | -0.05 | -0.14 | 0.02 | 0.38 |
| **Frequency × Predictability × Preview** | | | | | | | | |
| **FFD** | 6.86 | -2.67 | 16.08 | 0.84 | 0.14 | -0.04 | 0.33 | 1.37 |
| **SFD** | 0.91 | -9.26 | 11.29 | 0.37 | 0.10 | -0.11 | 0.30 | 0.65 |
| **GD** | 6.66 | -3.63 | 17.11 | 0.75 | 0.14 | -0.04 | 0.31 | 1.16 |
| **TT** | -2.40 | -15.69 | 11.10 | 0.50 | 0.03 | -0.12 | 0.19 | 0.35 |
| **Effects × Launch Distance:**<br>**Launch Distance** | | | | | | | | |
| **FFD** | 2.97 | -0.68 | 6.73 | 0.39 | **-0.18** | **-0.24** | **-0.12** | **>1000** |
| **SFD** | 2.07 | -2.17 | 6.23 | 0.23 | **-0.14** | **-0.22** | **-0.07** | **229.01** |
| **GD** | *5.05* | *0.58* | *9.48* | *1.66* | **-0.10** | **-0.16** | **-0.04** | **36.48** |
| **TT** | -0.70 | -5.91 | 4.46 | 0.20 | -0.04 | -0.09 | 0.01 | 0.36 |
| **Frequency × Launch Distance** | | | | | | | | |
| **FFD** | 0.35 | -5.00 | 5.72 | 0.17 | -0.07 | -0.17 | 0.03 | 0.54 |
| **SFD** | 0.15 | -5.84 | 6.05 | 0.20 | -0.03 | -0.15 | 0.09 | 0.30 |
| **GD** | -2.60 | -8.60 | 3.50 | 0.31 | *-0.11* | *-0.21* | *-0.02* | *2.89* |
| **TT** | -0.38 | -7.98 | 7.30 | 0.26 | -0.04 | -0.13 | 0.05 | 0.27 |
| **Predictability × Launch Distance** | | | | | | | | |
| **FFD** | 0.09 | -5.02 | 5.18 | 0.16 | -0.01 | -0.12 | 0.10 | 0.23 |
| **SFD** | -0.68 | -6.20 | 4.75 | 0.19 | 0.02 | -0.11 | 0.14 | 0.28 |
| **GD** | -0.69 | -6.22 | 4.80 | 0.19 | -0.03 | -0.13 | 0.07 | 0.27 |
| **TT** | 2.32 | -4.96 | 9.65 | 0.31 | 0.01 | -0.08 | 0.09 | 0.18 |
| **Preview × Launch Distance** | | | | | | | | |
| **FFD** | **36.46** | **31.01** | **42.06** | **>1000** | **0.50** | **0.39** | **0.61** | **>1000** |
| **SFD** | **35.98** | **29.87** | **42.32** | **>1000** | **0.44** | **0.32** | **0.57** | **>1000** |
| **GD** | **49.79** | **43.09** | **56.66** | **>1000** | **0.54** | **0.44** | **0.64** | **>1000** |
| **TT** | **53.72** | **44.36** | **63.26** | **>1000** | **0.33** | **0.24** | **0.42** | **>1000** |

| | | | | | | | | |
|---|---|---|---|---|---|---|---|---|
| **Frequency × Predictability × Launch Distance** | | | | | | | | |
| **FFD** | -1.72 | -11.36 | 8.05 | 0.33 | -0.08 | -0.27 | 0.12 | 0.57 |
| **SFD** | 2.49 | -7.89 | 12.96 | 0.40 | -0.06 | -0.29 | 0.16 | 0.53 |
| **GD** | 3.25 | -7.39 | 13.88 | 0.42 | 0.04 | -0.14 | 0.22 | 0.44 |
| **TT** | 0.65 | -12.63 | 13.86 | 0.47 | -0.03 | -0.18 | 0.13 | 0.34 |
| **Frequency × Preview × Launch Distance** | | | | | | | | |
| **FFD** | -5.40 | -15.47 | 4.57 | 0.56 | 0 | -0.19 | 0.18 | 0.41 |
| **SFD** | -6.40 | -16.74 | 3.74 | 0.74 | 0.01 | -0.20 | 0.22 | 0.45 |
| **GD** | -6.10 | -16.83 | 4.83 | 0.67 | 0.02 | -0.15 | 0.19 | 0.39 |
| **TT** | -6.64 | -20.36 | 6.97 | 0.73 | 0.03 | -0.12 | 0.18 | 0.35 |
| **Predictability × Preview × Launch Distance** | | | | | | | | |
| **FFD** | 4.83 | -4.34 | 14.19 | 0.50 | 0.10 | -0.09 | 0.28 | 0.70 |
| **SFD** | 6.68 | -3.23 | 16.42 | 0.87 | 0.11 | -0.10 | 0.32 | 0.76 |
| **GD** | 4.17 | -5.73 | 13.95 | 0.48 | 0.08 | -0.10 | 0.25 | 0.56 |
| **TT** | -2.05 | -15.13 | 11.12 | 0.49 | 0.01 | -0.14 | 0.16 | 0.33 |
| **Frequency × Predictability × Preview × Launch Distance** | | | | | | | | |
| **FFD** | 3.54 | -12.53 | 19.34 | 0.57 | 0.01 | -0.30 | 0.32 | 0.71 |
| **SFD** | -1.25 | -18.24 | 15.59 | 0.60 | 0.11 | -0.22 | 0.45 | 0.86 |
| **GD** | -0.23 | -17.32 | 16.71 | 0.61 | 0.03 | -0.25 | 0.33 | 0.62 |
| **TT** | -3.99 | -24.32 | 16.11 | 0.72 | 0.04 | -0.22 | 0.29 | 0.57 |

*Note*: Fixed effects on *mu* (central tendency) and on *beta* (skew) are presented in the left and right sets of columns, respectively. FFD = first fixation duration; SFD = single fixation duration; GD = gaze duration; TT = total fixation time. Effects with Credible Intervals (CrIs) not including zero and a $BF_{10}$ of more than 3 are highlighted in **bold**. Effects with CrIs not including zero or a $BF_{10}$ of more than 3, but not both, are *italicized*.

The results revealed very strong evidence for a Preview × Launch Distance interaction across all fixation duration measures ($BF_{10}$s > 1000). Launch distance affects the quality of a target's parafoveal preview, affording greater preview at shorter distances. When target preview was Valid, shorter launch distances produced faster fixations ($\Delta$Near–Far$_{(FFD, SFD, GD, TT)}$ = −21.2 [−25.6, −16.8], −20.1 [−25.1, −15.3], −30.0 [−35.2, −24.6], −26.1 [−32.5, −19.6] ms). Conversely, when target preview was Invalid, shorter launch distances slowed fixations ($\Delta$Near–Far$_{(FFD, SFD, GD, TT)}$ = 15.3 [10.4, 20.2], 15.9 [10.5, 21.3], 19.8 [13.6, 25.4], 27.5 [20.0, 34.9] ms). Launch distance modulated no other effects, confirming that the Frequency × Preview and Predictability × Preview interactions on GD persisted after accounting for launch distance.
